# Fault-tolerant adaptive attitude tracking control of satellite with uncertainty and actuator misalignment in presence of environmental disturbances

**DOI:** 10.1038/s41598-025-98468-0

**Published:** 2025-05-23

**Authors:** Mohammadjavad Hatami, Reza Nadafi, Mansour Kabganian

**Affiliations:** 1 Department of Mechanical Engineering, Amirkabir University of Technology, Tehran, 15875-4413 Iran; 2 Aerospace Research Institute, Amirkabir University of Technology, Tehran, 15875-4413 Iran

**Keywords:** Attitude tracking control, Uncertainty, Adaptive sliding mode control, Reaction wheel, Fault tolerant, Astronomy and planetary science, Engineering

## Abstract

In this study, a novel asymptotically stable attitude tracking controller is propounded for a satellite, operating in the existence of environmental disturbances, uncertain inertia matrix, reaction wheel misalignment, and actuator faults. Unlike existing methods, the proposed controller addresses multiple practical challenges simultaneously, including disturbances, uncertainties, misalignment, and faults. Incidentally a key advantage of the proposed controller is its ability to work without requiring a priori knowledge of the upper bound values of uncertainties and disturbances, which is a significant advancement over previous approaches. By deriving the kinematic and kinetic equations of the satellite system and defining appropriate sliding surfaces, the global asymptotic stability of the closed-loop system is guaranteed via Lyapunov theory. Simulation results, incorporating actuator saturation constraints, demonstrate the controller’s performance and robustness, achieving precise attitude tracking with 2% settling time of 7 s and saturation constraint of 0.12 Nm. Furthermore, a MATLAB Multibody simulation model validates the controller, yielding a maximum verification error less than 4% in angular velocity.

## Introduction

Attitude control is an indispensable aspect of any space mission. With advancements in space exploration, the attitude tracking problem has become a critical focus for satellites. Across a wide range of applications, including remote sensing, weather forecasting, telecommunication, navigation, technology demonstration, and some space activities such as rendezvous and docking^1^, attitude tracking is vital and plays a key role in mission success. Environmental disturbances, uncertainties, and actuator misalignments pose significant challenges to achieving high accuracy in satellite orientation. However, in some applications, precise attitude control is crucial. This necessity has led many researchers to extensively study satellite attitude tracking, making it a prominent field in recent years.

In the literature, several related studies have been conducted on spacecraft attitude tracking control. Cong et al.^2^ examined two sliding mode methods for spacecraft attitude tracking, addressing uncertainties in the inertia and environmental disturbances, assuming the known upper bound of these uncertainties. Jingrui et al.^3^ developed an attitude tracking controller for spacecraft using speed control moment gyros, considering uncertainties in the moment of inertia, gyro configuration, and rotor inertia. Hu et al.^4^ proposed an adaptive backstepping control strategy combined with a dynamic control allocation mechanism, accounting for actuator uncertainties and environmental disturbances. Their approach minimized energy consumption using optimal quadratic programming while adhering to actuator constraints. Gui et al.^5^ introduced an integral terminal sliding mode control strategy for rigid spacecraft, addressing actuator uncertainties such as faults and alignment errors, and conducted a comparative analysis of different control approaches. In a more recent study, Gui et al.^6^ presented a hybrid dual-quaternion integral sliding mode controller for combined position and attitude tracking of spacecraft. In a related study, Shen et al.^7^ focused on fault-tolerant attitude control with environmental disturbances, assuming the availability of known upper bounds for uncertainties and disturbances. Additionally, in another study, Shen et al.^8^ proposed an active fault-tolerant attitude control method that integrated actuator fault detection and handled control input constraints using a backstepping technique. Yue et al.^9^ designed a robust fault-tolerant attitude tracking controller with guaranteed performance, addressing issues such as actuator faults, misalignment, and sensor faults. Nadafi et al.^10^ introduced a robust control method for underactuated spacecraft, accounting for saturation constraints and time-varying perturbations. Their approach utilized a finite-time nonlinear disturbance observer-based backstepping technique to enhance robustness and demonstrated asymptotic stability even under a hard saturation limit of 0.035 N m. In another study by Nadafi et al.^11^ a super-twisting sliding mode controller designed with existence of saturation, inertia uncertainties and environmental disturbances. This study showed convergence to desired path with 0.08 N m saturation level within 230 s. Yang et al.^12^ investigated spacecraft attitude reorientation control, incorporating actuator faults, misalignment, and position constraints. They designed a reward function to manage position constraints and validated their approach through hardware-in-the-loop experiments. Su et al.^13^ developed an adaptive sliding mode controller for attitude tracking of a spacecraft without requiring initial condition information, stabilizing tracking errors within a small predefined area around the origin in a specified timeframe. Chen et al.^14^ brought up a nonsingular adaptive control strategy for on-orbit spacecraft orientation control, addressing actuator uncertainties, including failures and misalignment, while also exploring solutions for complete failure scenarios. Moreover, Zarourati et al.^15^ investigated underactuation faults in satellite reaction-wheel actuators during attitude tracking, emphasizing fault detection and diagnosis under nominal initial conditions. Their study introduced a finite-time sliding mode observer for fault detection. In another related work by Zarourati et al.^16^ a backstepping control method was developed to address the satellite attitude tracking problem, accounting for both underactuation faults and external disturbances. In another study, Jia et al.^17^ proposed a learning Chebyshev neural network-based approach for spacecraft attitude tracking control. Their work addressed uncertainties, actuator faults, and external disturbances in the spacecraft’s dynamic model. The proposed controller utilized a finite-time prescribed learning sliding mode control algorithm. Zhao et al.^18^ presented an adaptive neural-network-based control algorithm for spacecraft attitude tracking problem. This article used command filtered backstepping for generating control input addressing challenges such as actuator saturation, inertial uncertainty, and disturbances.

To conclude the limitations of previous studies based on the literature review, it can be observed that in some research, the upper bound values for disturbances or uncertainties are required, while these values are not always readily available. Additionally, few studies consider disturbances, uncertainties, faults, and misalignment simultaneously: most focus on addressing only some of these factors. Another limitation is the lack of attention to reaction wheels as actuators. Most studies treat system actuators in general terms without delving into specific details about them.

therefore, one of the primary challenges addressed in this study is the design of a robust attitude tracking controller that can effectively handle uncertainties and disturbances without requiring prior knowledge of their upper bound values. This is particularly challenging because most existing methods rely on predefined bounds, which are often unavailable in practical scenarios. Additionally, incorporating reaction wheel angular momentum as an input to the system introduces further complexity, as it requires careful consideration of actuator dynamics and constraints. These challenges are compounded by the need to simultaneously address environmental disturbances, uncertain inertia matrices, reaction wheel misalignment, and actuator faults, a combination that is rarely tackled in existing studies.

Therefore, the primary aim of this paper is to address mentioned limitations and improve the attitude tracking control for practical implementation. The main contributions of this study can be summarized as follows:

1. Comprehensive inclusion of factors: The controller design process incorporates environmental disturbances, satellite inertia uncertainty, reaction wheel misalignment, and actuator faults simultaneously. Input saturation is also considered in the simulation results.
2. Elimination of upper bound dependency: The proposed controller does not require knowledge of the upper bound values for environmental disturbances, uncertainties, misalignment, or actuator faults.

In this paper, sliding mode algorithm is considered for controller design because it is inherently robust to disturbances or faults and effective in dealing with real-world challenges^19^. Additionally, the proposed controller’s functionality is demonstrated through satellite modeling in MATLAB Multibody for verification analysis, where the controller’s performance is evaluated.

The remainder of the paper is organized as follows: section “Preliminaries and problem formulation” is dedicated to problem formulation and the derivation of the system’s kinematic and kinetic equations. Section “Controller design” presents the controller design. Simulation results for the proposed controller with analysis are demonstrated in section “Simulation results”. Finally, the conclusions of this study are provided in section “Conclusion”.

## Preliminaries and problem formulation

### Attitude dynamics

**Fig. 1:**
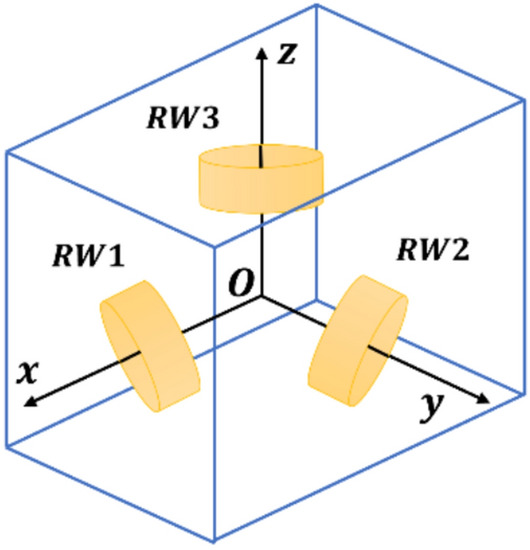
Configuration of reaction wheels in the body coordinate.

It is well-established that the total moment acting on a body about its center of mass equals the time rate change of the body’s angular momentum^20^ . This is a starting point to derive the kinetic equations of a satellite with reaction wheels. For a satellite with moment exchange devices, such as reaction wheels the momentum of the entire system consists of the momentum of satellite and the momentum of the reaction wheels. In this study we consider a satellite with three reaction wheels as depicted in Fig. 1. Thus, the attitude kinetics and kinematics of a satellite with reaction wheels can be derived as follows^20^:

1 $${\varvec{J}}\dot{{\varvec{\omega}}}+{{\varvec{\omega}}}^{\times }{\varvec{J}}{\varvec{\omega}}=-{\dot{{\varvec{h}}}}_{{\varvec{w}}}-{{\varvec{\omega}}}^{\times }{{\varvec{h}}}_{{\varvec{w}}}+{\varvec{d}}$$

2 $$\dot{\overline{{\varvec{\eta}}} }=\left[\begin{array}{c}{\dot{\eta }}_{0}\\ \dot{{\varvec{\eta}}}\end{array}\right]=\frac{1}{2}\left[\begin{array}{c}-{{\varvec{\eta}}}^{T}\\ {{\varvec{\eta}}}^{\times }+{\eta }_{0}{{\varvec{I}}}_{3}\end{array}\right]{\varvec{\omega}}$$

where ${\varvec{J}} \in {\mathbb{R}}^{3\times 3}$ states satellite inertia matrix specified in body-fixed frame, ${\varvec{\omega}}\in {\mathbb{R}}^{3}$ denotes the satellite’s angular velocity relative to an inertial frame, represented in body-fixed frame, ${\varvec{d}}\in {\mathbb{R}}^{3}$ represents the vector of environmental disturbances, $$\overline{{\varvec{\eta}} }={\left[\begin{array}{cc}{\eta }_{0}& {\eta }_{1}\end{array} \begin{array}{cc}{\eta }_{2}& {\eta }_{3}\end{array}\right]}^{T}={\left[\begin{array}{cc}{\eta }_{0}& {{\varvec{\eta}}}^{T}\end{array}\right]}^{T}\in {\mathbb{R}}\times {\mathbb{R}}^{3}$$ denotes quaternion parameters that describe the direction of body frame relative to an inertial frame and satisfies ${{\varvec{\eta}}}^{T}{\varvec{\eta}}+{\eta }_{0}=1$, ${{\varvec{h}}}_{{\varvec{w}}}\in {\mathbb{R}}^{3}$ is an angular momentum vector of reaction wheels, ${{\varvec{I}}}_{3}$ denotes $3\times 3$ identity matrix, the notation ${{\varvec{b}}}^{\times }$ for a vector $${\varvec{b}}={\left[\begin{array}{ccc}{b}_{1}& {b}_{2}& {b}_{3}\end{array}\right]}^{T}$$ is selected to specify skew-symmetric matrix. For the configuration of reaction wheels as demonstrated in Fig. 1, ${{\varvec{h}}}_{{\varvec{w}}}$ can be described as:

3 $${{\varvec{h}}}_{{\varvec{w}}}=\left[\begin{array}{c}{I}_{a}{\Omega }_{1}\\ {I}_{a}{\Omega }_{2}\\ {I}_{a}{\Omega }_{3}\end{array}\right]$$

Where ${I}_{a}\in {\mathbb{R}}$ is an axial moment of inertia of reaction wheel which is assumed identical for all wheels and ${\Omega }_{1},{\Omega }_{2},{\Omega }_{3}\in {\mathbb{R}}$ are the angular velocities of reaction wheels number 1 to 3, respectively. Moreover, the distance between the center of mass of each reaction wheel and the origin of the body coordinate is assumed to be the same.

### Reaction wheel misalignment

**Fig. 2:**
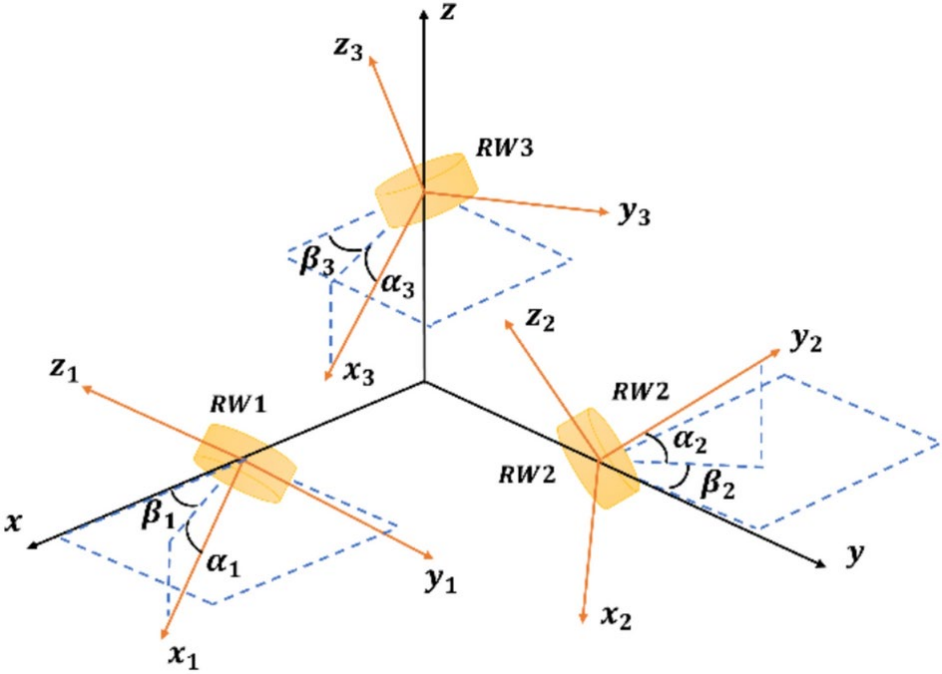
Satellite reaction wheels with misalignment.

In the process of assembling reaction wheels onto a satellite, it is impractical to mount them perfectly aligned. So, the misalignment effect of the reaction wheels should be considered in the dynamic equation of the satellite. Figure 2 illustrates the alignment errors of each reaction wheel. So, the Euler equation of the satellite (1) with actuator misalignment can be expressed as:

4 $${\varvec{J}}\dot{{\varvec{\omega}}}+{{\varvec{\omega}}}^{\times }{\varvec{J}}{\varvec{\omega}}=-{\varvec{D}}{\dot{{\varvec{h}}}}_{{\varvec{w}}}-{{\varvec{\omega}}}^{\times }{\varvec{D}}{{\varvec{h}}}_{{\varvec{w}}}+{\varvec{d}}$$

5 $${\varvec{D}}=\left[\begin{array}{c}{\cos}({\beta }_{1}){\cos}({\alpha }_{1})\\ {\sin}({\beta }_{1}){\cos}({\alpha }_{1})\\ -{\sin}({\alpha }_{1})\end{array} \begin{array}{c}-{\sin}({\beta }_{2}){\cos}({\alpha }_{2})\\ {\cos}({\beta }_{2}){\cos}({\alpha }_{2})\\ {\sin}({\alpha }_{2})\end{array} \begin{array}{c}{\cos}({\beta }_{3}){\sin}({\alpha }_{3})\\ {\sin}({\beta }_{3}){\sin}({\alpha }_{3})\\ {\cos}({\alpha }_{3})\end{array}\right]$$

Where ${\varvec{D}}\in {\mathbb{R}}^{3\times 3}$ is called misalignment matrix, and ${\alpha }_{i}$ and ${\beta }_{i}$ $(i \in \left\{{1,2},3\right\})$ are the deviation angles away from their aligned direction for reaction wheels 1 to 3, respectively. It is evident that if there is no misalignment in the satellite actuators, the misalignment matrix will be equal to identity matrix.

#### *Remark 1*

The existence of misalignment in satellite actuators alters the satellite inertia matrix, and this effect should be considered in controller design.

### Actuator fault

Faults in satellite actuators are inevitable, especially in reaction wheels. Reaction wheel faults can occur in the drive motor, bearings, and power supply due to insufficient lubrication or aging^8^. Therefore, actuator faults should be modeled and considered in the satellite’s dynamic equations. First, we define the nominal input to our system as:

6 $${{\varvec{u}}}_{{\varvec{c}}}=-{\dot{{\varvec{h}}}}_{{\varvec{w}}}$$

where ${\dot{{\varvec{h}}}}_{{\varvec{w}}}\in {\mathbb{R}}^{3}$ is reaction wheel torque vector, and ${{\varvec{u}}}_{{\varvec{c}}}\in {\mathbb{R}}^{3}$ is nominal torque vector. Second, according to previous studies^8^ fault model can be described as:

7 $${{\varvec{u}}}_{{\varvec{c}}}={\varvec{E}}{\varvec{u}}+\Delta {\varvec{u}}$$

where ${\varvec{E}}=diag\left\{{e}_{1},{e}_{2},{e}_{3}\right\}\in {\mathbb{R}}^{3\times 3}$ is called the effectiveness matrix that $0<{e}_{i}\le 1 (i\in \left\{{1,2},3\right\})$, ${\varvec{u}}\in {\mathbb{R}}^{3}$ denotes the actual moment acting on reaction wheels, $\Delta {\varvec{u}}\in {\mathbb{R}}^{3}$ is additive bias fault, and is assumed to be constant. So, the Euler equation of satellite (4) can be expressed as follows.

8 $${\varvec{J}}\dot{{\varvec{\omega}}}+{{\varvec{\omega}}}^{\times }({\varvec{J}}{\varvec{\omega}}+{\varvec{D}}{{\varvec{h}}}_{{\varvec{w}}})={\varvec{D}}({\varvec{E}}{\varvec{u}}+\Delta {\varvec{u}})+{\varvec{d}}$$

### Error dynamics

About tracking control of satellite attitude, the problem is brought up similar to the work^21^. Therefore, desired attitude of the satellite is expressed with a reference frame. The attitude of the reference frame is expressed through the quaternion ${\overline{{\varvec{\eta}}} }^{{\varvec{d}}}={\left[{\eta }_{0}^{d},{\left({{\varvec{\eta}}}^{{\varvec{d}}}\right)}^{T}\right]}^{T}$, which satisfies ${\left({{\varvec{\eta}}}^{{\varvec{d}}}\right)}^{T}{{\varvec{\eta}}}^{{\varvec{d}}}+{\left({\eta }_{0}^{d}\right)}^{2}=1$. Let ${{\varvec{\omega}}}_{{\varvec{d}}}\in {\mathbb{R}}^{3}$ be the desirable angular velocity of the reference frame.

#### Assumption 1

Desired angular velocity ${{\varvec{\omega}}}_{{\varvec{d}}}$ and derivative of that ${\dot{{\varvec{\omega}}}}_{{\varvec{d}}}$ are bounded for all $t\ge 0$. So, there are some constants as ${c}_{1}>0$ and ${c}_{2}>0$ which are finite such that $\Vert {{\varvec{\omega}}}_{{\varvec{d}}}\Vert \le {c}_{1}$ and $\Vert {\dot{{\varvec{\omega}}}}_{{\varvec{d}}}\Vert \le {c}_{2}$ for all $t\ge 0$.

Now, the quaternion error is defined as $\overline{{\varvec{q}} }={\left[{q}_{0},{{\varvec{q}}}^{T}\right]}^{T}$, which represents the attitude deviation of the body-fixed frame relative to the reference frame, fulfils ${{\varvec{q}}}^{T}{\varvec{q}}+{q}_{0}^{2}=1$. The relation between the quaternion error $\overline{{\varvec{q}} }$ to ${\overline{{\varvec{\eta}}} }^{{\varvec{d}}}$ and $\overline{{\varvec{\eta}} }$ is introduced as follows^22^:

9 $$\overline{{\varvec{q}} }=\left[\begin{array}{c}{\eta }_{0}^{d}{\eta }_{0}+{\left({{\varvec{\eta}}}^{{\varvec{d}}}\right)}^{T}\eta \\ {\eta }_{0}^{d}\eta -{\eta }_{0}{{\varvec{\eta}}}^{{\varvec{d}}}-{\left({{\varvec{\eta}}}^{{\varvec{d}}}\right)}^{\times }\eta \end{array}\right]$$

The angular velocity error is then expressed by:

10 $$\widetilde{{\varvec{\omega}}}={\varvec{\omega}}-{\varvec{R}}\left(\overline{{\varvec{q}} }\right){{\varvec{\omega}}}_{{\varvec{d}}}$$

where ${\varvec{R}}(\overline{{\varvec{q}} })\in {\mathbb{R}}^{3\times 3}$ denotes the rotation matrix that transforms coordinates from the reference frame to the body-fixed frame and is specified by^23^:

11 $${\varvec{R}}\left(\overline{{\varvec{q}} }\right)=\left({q}_{0}^{2}-{{\varvec{q}}}^{T}{\varvec{q}}\right){{\varvec{I}}}_{3}+2{\varvec{q}}{{\varvec{q}}}^{T}-2{q}_{0}{{\varvec{q}}}^{\times }$$

It follows from^24^ that ${{\varvec{R}}}^{T}{\varvec{R}}=1$ and $\dot{{\varvec{R}}}\left(\overline{{\varvec{q}} }\right)=-{\widetilde{{\varvec{\omega}}}}^{\times }{\varvec{R}}(\overline{{\varvec{q}} })$. So, from the angular velocity error of the satellite (10) and the dynamic equation (8), we can express the attitude error and the kinematics of the satellite as follows:

12 $${\varvec{J}}\dot{\widetilde{{\varvec{\omega}}}}={\varvec{D}}\left({\varvec{E}}{\varvec{u}}+\Delta {\varvec{u}}\right)+{\varvec{d}}-{{\varvec{\omega}}}^{\times }\left({\varvec{J}}{\varvec{\omega}}+{\varvec{D}}{{\varvec{h}}}_{{\varvec{w}}}\right)+{\varvec{J}}({\widetilde{{\varvec{\omega}}}}^{\times }{\varvec{R}}\left(\overline{{\varvec{q}} }\right){{\varvec{\omega}}}_{{\varvec{d}}}-{\varvec{R}}\left(\overline{{\varvec{q}} }\right){{\dot{{\varvec{\omega}}}}_{{\varvec{d}}}})$$

13 $$\dot{\overline{{\varvec{q}}} }=\left[\begin{array}{c}{\dot{q}}_{0}\\ \dot{{\varvec{q}}}\end{array}\right]=\frac{1}{2}\left[\begin{array}{c}-{{\varvec{q}}}^{T}\\ {{\varvec{q}}}^{\times }+{q}_{0}{{\varvec{I}}}_{3}\end{array}\right]\widetilde{{\varvec{\omega}}}$$

### Problem statement

The objective of this paper is to develop an adaptive sliding mode controller in order to $\underset{t\to \infty }{\text{lim}}{\varvec{q}}(t)\to 0$ and $\underset{t\to \infty }{\text{lim}}\widetilde{{\varvec{\omega}}}(t)\to 0$. It can be resulted that problem of attitude tracking is the same as stabilization for $\widetilde{{\varvec{\omega}}}$ and ${\varvec{q}}$.

## Controller design

In this section, an adaptive control law is designed for tracking the satellite’s attitude with environmental disturbances, inertia uncertainty, actuator misalignment, and faults. First, a sliding surface is introduced. Subsequently, a control input is formulated. Eventually, a smooth law is expressed to stay away from chattering in the satellite input.

For facilitating controller design, we should consider the following assumptions for our system.

### Assumption 2

Consider ${\varvec{J}}=\overline{{\varvec{J}} }+\Delta {\varvec{J}}$ that $\overline{{\varvec{J}} }$ and $\Delta {\varvec{J}}$ are available and uncertain part of the satellite inertia respectively. We assume $\Vert \Delta {\varvec{J}}\Vert \le {\gamma }_{0}.$

### Assumption 3

Consider ${\varvec{D}}=\overline{{\varvec{D}} }+\Delta \mathbf{D}$ and ${\varvec{E}}=\overline{{\varvec{E}} }+\Delta {\varvec{E}}$ so that $\overline{{\varvec{D}} }$ and $\overline{{\varvec{E}} }$ are known parts of misalignment and effectiveness matrices respectively. $\Delta {\varvec{D}}$ and $\Delta {\varvec{E}}$ are unknown parts of mentioned matrices which satisfies $\Vert \Delta {\varvec{D}}\Vert \le {\gamma }_{1}, \Vert \Delta {\varvec{E}}\Vert \le {\gamma }_{2}.$

### Assumption 4

All environmental disturbances, including those caused by gravitational forces, magnetic forces, and solar radiation, are considered to be limited. Additionally, we assume proportionality of the aerodynamic drag with the square of the angular velocity^25^. the environmental disturbances ${\varvec{d}}$ are considered to be $\Vert {\varvec{d}}\Vert \le {\upgamma }_{3}+{\gamma }_{4}{\Vert \widetilde{{\varvec{\omega}}}\Vert }^{2}.$

### Assumption 5

The satellite’s control law may incorporate the error of angular velocity and the quaternion error to address the attitude control problem, with the quaternion error being inherently bounded. Thus, we consider that $\Vert {\varvec{u}}\Vert \le {\gamma }_{7}+{\gamma }_{8}\Vert \widetilde{{\varvec{\omega}}}\Vert +{\gamma }_{9}{\Vert \widetilde{{\varvec{\omega}}}\Vert }^{2}$.

### Assumption 6

Reaction wheels are precise equipment that their technical information is available. So, at this study, we assume that axial moment of inertia of the reaction wheels ${I}_{a}$ is known and we have no uncertainty in it.

In order to design sliding mode controller, we consider sliding surface as below:

14 $${\varvec{s}}=b(\widetilde{{\varvec{\omega}}}+{\varvec{C}}{\varvec{q}})$$

where scalar $b>0$ and ${{\varvec{C}}}_{3\times 3}$ which is positive definite constant matrix are control gains for attitude tracking. We derived the error dynamic equation of our satellite (12) in the previous section. Now, by applying the above assumptions, we can rewrite it as below.

15 $$(\overline{{\varvec{J}} }+\Delta {\varvec{J}})\dot{\widetilde{{\varvec{\omega}}}}=(\overline{{\varvec{D}} }+\Delta {\varvec{D}})\left(\left(\overline{{\varvec{E}} }+\Delta {\varvec{E}}\right){\varvec{u}}+\Delta {\varvec{u}}\right)+{\varvec{d}}-{{\varvec{\omega}}}^{\times }\left(\left(\overline{{\varvec{J}} }+\Delta {\varvec{J}}\right){\varvec{\omega}}+\left(\overline{{\varvec{D}} }+\Delta {\varvec{D}}\right){{\varvec{h}}}_{{\varvec{w}}}\right)+(\overline{{\varvec{J}} }+\Delta {\varvec{J}})({\widetilde{{\varvec{\omega}}}}^{\times }{\varvec{R}}\left(\overline{{\varvec{q}} }\right){{\varvec{\omega}}}_{{\varvec{d}}}-{\varvec{R}}\left(\overline{{\varvec{q}} }\right){{\dot{{\varvec{\omega}}}}_{{\varvec{d}}}})$$

16 $$\begin{aligned}\overline{{\varvec{J}}}\dot{\widetilde{{\varvec{\omega}}} }+\Delta {\varvec{J}}\dot{\widetilde{{\varvec{\omega}}}}&=\left(\overline{{\varvec{D}} }+\Delta {\varvec{D}}\right)\left(\overline{{\varvec{E}}}{\varvec{u} }+{\varvec{u}}\Delta {\varvec{E}}+\Delta {\varvec{u}}\right)+{\varvec{d}}-{{\varvec{\omega}}}^{\times }\left(\overline{{\varvec{J}}}{\varvec{\omega} }+{\varvec{\omega}}\Delta {\varvec{J}}+\overline{{\varvec{D}}}{{\varvec{h}} }_{{\varvec{w}}}+{{\varvec{h}}}_{{\varvec{w}}}\Delta {\varvec{D}}\right)\\ &\quad+\overline{{\varvec{J}} }\left({\widetilde{{\varvec{\omega}}}}^{\times }{\varvec{R}}\left(\overline{{\varvec{q}} }\right){{\varvec{\omega}}}_{{\varvec{d}}}-{\varvec{R}}\left(\overline{{\varvec{q}} }\right){{\dot{{\varvec{\omega}}}}_{{\varvec{d}}}}\right)+\Delta {\varvec{J}}({\widetilde{{\varvec{\omega}}}}^{\times }{\varvec{R}}\left(\overline{{\varvec{q}} }\right){{\varvec{\omega}}}_{{\varvec{d}}}-{\varvec{R}}\left(\overline{{\varvec{q}} }\right){{\dot{{\varvec{\omega}}}}_{{\varvec{d}}}})\end{aligned}$$

17 $$\begin{aligned}\overline{{\varvec{J}}}\dot{\widetilde{{\varvec{\omega}}} }+\Delta {\varvec{J}}\dot{\widetilde{{\varvec{\omega}}}} &=\overline{{\varvec{D}} }\left(\overline{{\varvec{E}}}{\varvec{u} }+{\varvec{u}}\Delta {\varvec{E}}+\Delta {\varvec{u}}\right)+\Delta {\varvec{D}}\left(\overline{{\varvec{E}}}{\varvec{u} }+{\varvec{u}}\Delta {\varvec{E}}+\Delta {\varvec{u}}\right)+{\varvec{d}}-{{\varvec{\omega}}}^{\times }\left(\overline{{\varvec{J}}}{\varvec{\omega} }+\overline{{\varvec{D}}}{{\varvec{h}} }_{{\varvec{w}}}\right)\\ &\quad-{{\varvec{\omega}}}^{\times }\left({\varvec{\omega}}\Delta {\varvec{J}}+{{\varvec{h}}}_{{\varvec{w}}}\Delta {\varvec{D}}\right)+\overline{{\varvec{J}} }\left({\widetilde{{\varvec{\omega}}}}^{\times }{\varvec{R}}\left(\overline{{\varvec{q}} }\right){{\varvec{\omega}}}_{{\varvec{d}}}-{\varvec{R}}\left(\overline{{\varvec{q}} }\right){{\dot{{\varvec{\omega}}}}_{{\varvec{d}}}}\right)+\Delta {\varvec{J}}({\widetilde{{\varvec{\omega}}}}^{\times }{\varvec{R}}\left(\overline{{\varvec{q}} }\right){{\varvec{\omega}}}_{{\varvec{d}}}-{\varvec{R}}\left(\overline{{\varvec{q}} }\right){{\dot{{\varvec{\omega}}}}_{{\varvec{d}}}})\end{aligned}$$

18 $$\begin{aligned}\overline{{\varvec{J}}}\dot{\widetilde{{\varvec{\omega}}} }+\Delta {\varvec{J}}\dot{\widetilde{{\varvec{\omega}}}}&=\overline{{\varvec{D}} }\overline{{\varvec{E}}}{\varvec{u} }+\overline{{\varvec{D}} }\left({\varvec{u}}\Delta {\varvec{E}}+\Delta {\varvec{u}}\right)+\Delta {\varvec{D}}\left(\overline{{\varvec{E}}}{\varvec{u} }+{\varvec{u}}\Delta {\varvec{E}}+\Delta {\varvec{u}}\right)+{\varvec{d}}-{{\varvec{\omega}}}^{\times }\left(\overline{{\varvec{J}}}{\varvec{\omega} }+\overline{{\varvec{D}}}{{\varvec{h}} }_{{\varvec{w}}}\right)\\ &\quad-{{\varvec{\omega}}}^{\times }\left({\varvec{\omega}}\Delta {\varvec{J}}+{{\varvec{h}}}_{{\varvec{w}}}\Delta {\varvec{D}}\right)+\overline{{\varvec{J}} }\left({\widetilde{{\varvec{\omega}}}}^{\times }{\varvec{R}}\left(\overline{{\varvec{q}} }\right){{\varvec{\omega}}}_{{\varvec{d}}}-{\varvec{R}}\left(\overline{{\varvec{q}} }\right){{\dot{{\varvec{\omega}}}}_{{\varvec{d}}}}\right)+\Delta {\varvec{J}}({\widetilde{{\varvec{\omega}}}}^{\times }{\varvec{R}}\left(\overline{{\varvec{q}} }\right){{\varvec{\omega}}}_{{\varvec{d}}}-{\varvec{R}}\left(\overline{{\varvec{q}} }\right){{\dot{{\varvec{\omega}}}}_{{\varvec{d}}}})\end{aligned}$$

19 $$\begin{aligned}\overline{{\varvec{J}}}\dot{\widetilde{{\varvec{\omega}}} }&=\overline{{\varvec{D}} }\overline{{\varvec{E}}}{\varvec{u} }-{{\varvec{\omega}}}^{\times }\left(\overline{{\varvec{J}}}{\varvec{\omega} }+\overline{{\varvec{D}}}{{\varvec{h}} }_{{\varvec{w}}}\right)+\overline{{\varvec{J}} }\left({\widetilde{{\varvec{\omega}}}}^{\times }{\varvec{R}}\left(\overline{{\varvec{q}} }\right){{\varvec{\omega}}}_{{\varvec{d}}}-{\varvec{R}}\left(\overline{{\varvec{q}} }\right){{\dot{{\varvec{\omega}}}}_{{\varvec{d}}}}\right)+(-\Delta {\varvec{J}}\dot{\widetilde{{\varvec{\omega}}}}+\overline{{\varvec{D}} }\left({\varvec{u}}\Delta {\varvec{E}}+\Delta {\varvec{u}}\right)\\ &\quad+\Delta {\varvec{D}}\left(\overline{{\varvec{E}}}{\varvec{u} }+{\varvec{u}}\Delta {\varvec{E}}+\Delta {\varvec{u}}\right)+{\varvec{d}}-{{\varvec{\omega}}}^{\times }\left({\varvec{\omega}}\Delta {\varvec{J}}+{{\varvec{h}}}_{{\varvec{w}}}\Delta {\varvec{D}}\right)+\Delta {\varvec{J}}({\widetilde{{\varvec{\omega}}}}^{\times }{\varvec{R}}\left(\overline{{\varvec{q}} }\right){{\varvec{\omega}}}_{{\varvec{d}}}-{\varvec{R}}\left(\overline{{\varvec{q}} }\right){{\dot{{\varvec{\omega}}}}_{{\varvec{d}}}}))\end{aligned}$$

20 $$\begin{aligned}\dot{\widetilde{{\varvec{\omega}}}}&={\overline{{\varvec{J}}} }^{-1}\overline{{\varvec{D}} }\overline{{\varvec{E}}}{\varvec{u} }-{\overline{{\varvec{J}}} }^{-1}{{\varvec{\omega}}}^{\times }\left(\overline{{\varvec{J}}}{\varvec{\omega} }+\overline{{\varvec{D}}}{{\varvec{h}} }_{{\varvec{w}}}\right)+\left({\widetilde{{\varvec{\omega}}}}^{\times }{\varvec{R}}\left(\overline{{\varvec{q}} }\right){{\varvec{\omega}}}_{{\varvec{d}}}-{\varvec{R}}\left(\overline{{\varvec{q}} }\right){{\dot{{\varvec{\omega}}}}_{{\varvec{d}}}}\right)+{\overline{{\varvec{J}}} }^{-1}(-\Delta {\varvec{J}}\dot{\widetilde{{\varvec{\omega}}}}+\overline{{\varvec{D}} }\left({\varvec{u}}\Delta {\varvec{E}}+\Delta {\varvec{u}}\right)\\ &\quad+\Delta {\varvec{D}}\left(\overline{{\varvec{E}}}{\varvec{u} }+{\varvec{u}}\Delta {\varvec{E}}+\Delta {\varvec{u}}\right)+{\varvec{d}}-{{\varvec{\omega}}}^{\times }\left({\varvec{\omega}}\Delta {\varvec{J}}+{{\varvec{h}}}_{{\varvec{w}}}\Delta {\varvec{D}}\right)+\Delta {\varvec{J}}({\widetilde{{\varvec{\omega}}}}^{\times }{\varvec{R}}\left(\overline{{\varvec{q}} }\right){{\varvec{\omega}}}_{{\varvec{d}}}-{\varvec{R}}\left(\overline{{\varvec{q}} }\right){{\dot{{\varvec{\omega}}}}_{{\varvec{d}}}}))\end{aligned}$$

By considering ${\varvec{Q}}=-{\overline{{\varvec{J}}} }^{-1}{{\varvec{\omega}}}^{\times }\left(\overline{{\varvec{J}}}{\varvec{\omega} }+\overline{{\varvec{D}}}{{\varvec{h}} }_{{\varvec{w}}}\right)+\left({\widetilde{{\varvec{\omega}}}}^{\times }{\varvec{R}}\left(\overline{{\varvec{q}} }\right){{\varvec{\omega}}}_{{\varvec{d}}}-{\varvec{R}}\left(\overline{{\varvec{q}} }\right){{\dot{{\varvec{\omega}}}}_{{\varvec{d}}}}\right)+\frac{1}{2}{\varvec{C}}({{\varvec{q}}}^{\times }+{q}_{0}{{\varvec{I}}}_{3})\widetilde{{\varvec{\omega}}}$ and ${\varvec{\rho}}={\overline{{\varvec{J}}} }^{-1}(-\Delta {\varvec{J}}\dot{\widetilde{{\varvec{\omega}}}}+\overline{{\varvec{D}} }\left({\varvec{u}}\Delta {\varvec{E}}+\Delta {\varvec{u}}\right)+\Delta {\varvec{D}}\left(\overline{{\varvec{E}}}{\varvec{u} }+{\varvec{u}}\Delta {\varvec{E}}+\Delta {\varvec{u}}\right)+{\varvec{d}}-{{\varvec{\omega}}}^{\times }\left({\varvec{\omega}}\Delta {\varvec{J}}+{{\varvec{h}}}_{{\varvec{w}}}\Delta {\varvec{D}}\right)+\Delta {\varvec{J}}({\widetilde{{\varvec{\omega}}}}^{\times }{\varvec{R}}\left(\overline{{\varvec{q}} }\right){{\varvec{\omega}}}_{{\varvec{d}}}-{\varvec{R}}\left(\overline{{\varvec{q}} }\right){{\dot{{\varvec{\omega}}}}_{{\varvec{d}}}}))$, we can obtain a compact expression for the attitude error dynamic as follows:

21 $$\dot{\widetilde{{\varvec{\omega}}}}+{\varvec{C}}\dot{{\varvec{q}}}={\varvec{Q}}+{\varvec{\rho}}+{\overline{{\varvec{J}}} }^{-1}\overline{{\varvec{D}} }\overline{{\varvec{E}}}{\varvec{u} }$$

In the error dynamic equation (21), ${\varvec{\rho}}$ contains all uncertainties that, under equation (12) and assumption 5, we can easily verify that ${\varvec{\rho}}$ is bounded by following function:

22 $${\Vert {\varvec{\rho}}\Vert }_{1}\le {d}_{0}+{d}_{1}{\Vert \widetilde{{\varvec{\omega}}}\Vert }_{1}+{d}_{2}{\Vert \widetilde{{\varvec{\omega}}}\Vert }_{1}^{2}$$

The control input to our system is considered as:

23 $${\varvec{u}}=-{\overline{{\varvec{E}}} }^{-1}{\overline{{\varvec{D}}} }^{-1}\overline{{\varvec{J}} }({\varvec{Q}}+{\varvec{K}}{\varvec{s}}+\widehat{\alpha }sgn({\varvec{s}}))$$

where ${{\varvec{K}}}_{3\times 3}$ is gain matrix which is positive definite, $\widehat{\alpha }={\widehat{d}}_{0}+{\widehat{d}}_{1}{\Vert \widetilde{{\varvec{\omega}}}\Vert }_{1}+{\widehat{d}}_{2}{\Vert \widetilde{{\varvec{\omega}}}\Vert }_{1}^{2}$ that ${\widehat{d}}_{0}, {\widehat{d}}_{1}, {\widehat{d}}_{2}$ are estimations of ${d}_{0},{d}_{1}, {d}_{2}$ respectively and estimation errors are defined as ${\widetilde{d}}_{0}={\widehat{d}}_{0}-{d}_{0}$, ${\widetilde{d}}_{1}={\widehat{d}}_{1}-{d}_{1}$ and ${\widetilde{d}}_{2}={\widehat{d}}_{2}-{d}_{2}$. Estimated parameters are derived under following adaptation laws.

24 $${\dot{\widehat{d}}}_{0}={k}_{0}{\Vert {\varvec{s}}\Vert }_{1}$$

25 $${\dot{\widehat{d}}}_{1}={k}_{1}{\Vert {\varvec{s}}\Vert }_{1}{\Vert \widetilde{{\varvec{\omega}}}\Vert }_{1}$$

26 $${\dot{\widehat{d}}}_{2}={k}_{2}{\Vert {\varvec{s}}\Vert }_{1}{\Vert \widetilde{{\varvec{\omega}}}\Vert }_{1}^{2}$$

where ${k}_{0}, {k}_{1}, {k}_{2}$ are positive adaptive gains. Note that since ${d}_{0}, {d}_{1}, {d}_{2}$ are constant parameters then ${\dot{\widetilde{d}}}_{i}={\dot{\widehat{d}}}_{i} (i\in \left\{{1,2},3\right\})$. As a reminder $$sgn\left({\varvec{s}}\right)={\left[\begin{array}{ccc}sgn({s}_{11})& sgn({s}_{21})& sgn({s}_{31})\end{array}\right]}^{T}$$ denotes as a sign function.

### *Remark 2*

In the introduced control input (23), $\overline{{\varvec{E}} }, \overline{{\varvec{D}} }, \overline{{\varvec{J}} }$ are known positive definite matrices and invertible.

### **Lemma 1**

*Consider the satellite dynamic equation and the error dynamic equation in* $\left(12\right), \left(21\right)$ *and also the sliding surface in* (14). *If control input in* (23) *is applied to the system, then* $\underset{t\to \infty }{\text{lim}}{\varvec{q}}(t)\to 0$ *and* $\underset{t\to \infty }{\text{lim}}\widetilde{{\varvec{\omega}}}(t)\to 0$ *which means that the attitude tracking errors converge to zero asymptotically and tracking objective is obtained*.

### *Proof*

The following Lyapunov candidate function which is positive definite is selected.

27 $$V=\frac{1}{2}{{\varvec{s}}}^{T}{b}^{-1}{\varvec{s}}+\frac{1}{2}{k}_{0}^{-1}{\widetilde{d}}_{0}^{2}+\frac{1}{2}{k}_{1}^{-1}{\widetilde{d}}_{1}^{2}+\frac{1}{2}{k}_{2}^{-1}{\widetilde{d}}_{2}^{2}$$

The time derivative of $V$ is as below.

28 $$\dot{V}=\frac{1}{2}{\dot{{\varvec{s}}}}^{T}{b}^{-1}{\varvec{s}}+\frac{1}{2}{{\varvec{s}}}^{T}{b}^{-1}\dot{{\varvec{s}}}+{k}_{0}^{-1}{\dot{\widetilde{d}}}_{0}{\widetilde{d}}_{0}+{k}_{1}^{-1}{\dot{\widetilde{d}}}_{1}{\widetilde{d}}_{1}+{k}_{2}^{-1}{\dot{\widetilde{d}}}_{2}{\widetilde{d}}_{2}$$

29 $$\dot{V}={{\varvec{s}}}^{T}{b}^{-1}\dot{{\varvec{s}}}+{k}_{0}^{-1}{\dot{\widetilde{d}}}_{0}{\widetilde{d}}_{0}+{k}_{1}^{-1}{\dot{\widetilde{d}}}_{1}{\widetilde{d}}_{1}+{k}_{2}^{-1}{\dot{\widetilde{d}}}_{2}{\widetilde{d}}_{2}$$

30 $$\dot{V}={{\varvec{s}}}^{T}{b}^{-1}b\left(\dot{\widetilde{{\varvec{\omega}}}}+{\varvec{C}}\dot{{\varvec{q}}}\right)+{k}_{0}^{-1}{\dot{\widetilde{d}}}_{0}{\widetilde{d}}_{0}+{k}_{1}^{-1}{\dot{\widetilde{d}}}_{1}{\widetilde{d}}_{1}+{k}_{2}^{-1}{\dot{\widetilde{d}}}_{2}{\widetilde{d}}_{2}$$

31 $$\dot{V}={{\varvec{s}}}^{T}\left({\varvec{Q}}+{\varvec{\rho}}+{\overline{{\varvec{J}}} }^{-1}\overline{{\varvec{D}} }\overline{{\varvec{E}}}{\varvec{u} }\right)+{k}_{0}^{-1}{\dot{\widetilde{d}}}_{0}{\widetilde{d}}_{0}+{k}_{1}^{-1}{\dot{\widetilde{d}}}_{1}{\widetilde{d}}_{1}+{k}_{2}^{-1}{\dot{\widetilde{d}}}_{2}{\widetilde{d}}_{2}$$

32 $$\dot{V}={{\varvec{s}}}^{T}\left(-{\varvec{K}}{\varvec{s}}-\widehat{\alpha }sgn\left({\varvec{s}}\right)+{\varvec{\rho}}\right)+{k}_{0}^{-1}{\dot{\widetilde{d}}}_{0}{\widetilde{d}}_{0}+{k}_{1}^{-1}{\dot{\widetilde{d}}}_{1}{\widetilde{d}}_{1}+{k}_{2}^{-1}{\dot{\widetilde{d}}}_{2}{\widetilde{d}}_{2}$$

33 $$\dot{V}=-{{\varvec{s}}}^{T}{\varvec{K}}{\varvec{s}}-\widehat{\alpha }{\Vert {\varvec{s}}\Vert }_{1}+{{\varvec{s}}}^{T}{\varvec{\rho}}+{k}_{0}^{-1}{\dot{\widetilde{d}}}_{0}{\widetilde{d}}_{0}+{k}_{1}^{-1}{\dot{\widetilde{d}}}_{1}{\widetilde{d}}_{1}+{k}_{2}^{-1}{\dot{\widetilde{d}}}_{2}{\widetilde{d}}_{2}$$

Now in view of $\left(22\right), \left(24\right), \left(25\right), (26)$ we have:

34 $$\dot{V}\le -{{\varvec{s}}}^{T}{\varvec{K}}{\varvec{s}}-\widehat{\alpha }{\Vert {\varvec{s}}\Vert }_{1}+{\Vert {\varvec{s}}\Vert }_{1}{\Vert {\varvec{\rho}}\Vert }_{1}+{\Vert {\varvec{s}}\Vert }_{1}{\widetilde{d}}_{0}+{\Vert {\varvec{s}}\Vert }_{1}{\Vert \widetilde{{\varvec{\omega}}}\Vert }_{1}{\widetilde{d}}_{1}+{\Vert {\varvec{s}}\Vert }_{1}{\Vert \widetilde{{\varvec{\omega}}}\Vert }_{1}^{2}{\widetilde{d}}_{2}$$

35 $$\dot{V}\le -{{\varvec{s}}}^{T}{\varvec{K}}{\varvec{s}}-({\widehat{d}}_{0}+{\widehat{d}}_{1}{\Vert \widetilde{{\varvec{\omega}}}\Vert }_{1}+{\widehat{d}}_{2}{\Vert \widetilde{{\varvec{\omega}}}\Vert }_{1}^{2}){\Vert {\varvec{s}}\Vert }_{1}+{\Vert {\varvec{s}}\Vert }_{1}{\Vert {\varvec{\rho}}\Vert }_{1}+{\Vert {\varvec{s}}\Vert }_{1}{\widetilde{d}}_{0}+{\Vert {\varvec{s}}\Vert }_{1}{\Vert \widetilde{{\varvec{\omega}}}\Vert }_{1}{\widetilde{d}}_{1}+{\Vert {\varvec{s}}\Vert }_{1}{\Vert \widetilde{{\varvec{\omega}}}\Vert }_{1}^{2}{\widetilde{d}}_{2}$$

36 $$\dot{V}\le -{{\varvec{s}}}^{T}{\varvec{K}}{\varvec{s}}-({\widehat{d}}_{0}+{\widehat{d}}_{1}{\Vert \widetilde{{\varvec{\omega}}}\Vert }_{1}+{\widehat{d}}_{2}{\Vert \widetilde{{\varvec{\omega}}}\Vert }_{1}^{2}){\Vert {\varvec{s}}\Vert }_{1}+{\Vert {\varvec{s}}\Vert }_{1}({d}_{0}+{d}_{1}{\Vert \widetilde{{\varvec{\omega}}}\Vert }_{1}+{d}_{2}{\Vert \widetilde{{\varvec{\omega}}}\Vert }_{1}^{2})+{\Vert {\varvec{s}}\Vert }_{1}{\widetilde{d}}_{0}+{\Vert {\varvec{s}}\Vert }_{1}{\Vert \widetilde{{\varvec{\omega}}}\Vert }_{1}{\widetilde{d}}_{1}+{\Vert {\varvec{s}}\Vert }_{1}{\Vert \widetilde{{\varvec{\omega}}}\Vert }_{1}^{2}{\widetilde{d}}_{2}$$

37 $$\dot{V}\le -{{\varvec{s}}}^{T}{\varvec{K}}{\varvec{s}}-{\Vert {\varvec{s}}\Vert }_{1}\left({\widetilde{d}}_{0}+{\widetilde{d}}_{1}{\Vert \widetilde{{\varvec{\omega}}}\Vert }_{1}+{\widetilde{d}}_{2}{\Vert \widetilde{{\varvec{\omega}}}\Vert }_{1}^{2}\right)+{\Vert {\varvec{s}}\Vert }_{1}{\widetilde{d}}_{0}+{\Vert {\varvec{s}}\Vert }_{1}{\Vert \widetilde{{\varvec{\omega}}}\Vert }_{1}{\widetilde{d}}_{1}+{\Vert {\varvec{s}}\Vert }_{1}{\Vert \widetilde{{\varvec{\omega}}}\Vert }_{1}^{2}{\widetilde{d}}_{2}$$

38 $$\dot{V}\le -{{\varvec{s}}}^{T}{\varvec{K}}{\varvec{s}}$$

Therefore, by computing $\ddot{V}$, its boundedness can easily be shown. Moreover, $\widetilde{{\varvec{\omega}}}$ is bounded through its definition. So, with continuity of $\dot{V}$ according to the Barbalat’s lemma^26^, we can prove that error dynamic equation (21) is globally asymptotically stable, which means $\underset{t\to \infty }{\text{lim}}{\varvec{q}}(t)\to 0$ and $\underset{t\to \infty }{\text{lim}}\widetilde{{\varvec{\omega}}}(t)\to 0$.

The proposed control law exhibits chattering due to the sign function. To mitigate chattering in the satellite input, the sign function can be substituted with a saturation function, which is defined as:

39 $$sat\left(x\right)=\left\{\begin{array}{ll}sign\left(x\right),&\quad if \left|x\right|>\varphi \\ \frac{x}{\varphi },&\quad if \left|x\right|\le \varphi \end{array}\right.$$

where $\varphi$ is boundary layer thickness. It is important to note that the estimated gains may become unlimited within the boundary layer^25^. To address this issue, the designed control law is modified as below.

40 $${\varvec{u}}=-{\overline{{\varvec{E}}} }^{-1}{\overline{{\varvec{D}}} }^{-1}\overline{{\varvec{J}} }({\varvec{Q}}+{\varvec{K}}\overline{{\varvec{s}} }+\widehat{\alpha }sat({\varvec{s}}))$$

where $$sat\left({\varvec{s}}\right)={\left[\begin{array}{ccc}sat({s}_{11})& sat({s}_{21})& sat({s}_{31})\end{array}\right]}^{T}$$ and $\overline{{\varvec{s}} }={\varvec{s}}-\varphi sat({\varvec{s}})$ shows distance between boundary layer and the current state^27^. Therefore, the adaptation laws should be altered as follows either.

41 $${\dot{\widehat{d}}}_{0}={k}_{0}{\Vert \overline{{\varvec{s}}}\Vert }_{1}$$

42 $${\dot{\widehat{d}}}_{1}={k}_{1}{\Vert \overline{{\varvec{s}}}\Vert }_{1}{\Vert \widetilde{{\varvec{\omega}}}\Vert }_{1}$$

43 $${\dot{\widehat{d}}}_{2}={k}_{2}{\Vert \overline{{\varvec{s}}}\Vert }_{1}{\Vert \widetilde{{\varvec{\omega}}}\Vert }_{1}^{2}$$

In this case, the following Lyapunov candidate function can be utilized.

44 $$\overline{V }=\frac{1}{2}{\overline{{\varvec{s}}} }^{T}{b}^{-1}\overline{{\varvec{s}} }+\frac{1}{2}{k}_{0}^{-1}{\widetilde{d}}_{0}^{2}+\frac{1}{2}{k}_{1}^{-1}{\widetilde{d}}_{1}^{2}+\frac{1}{2}{k}_{2}^{-1}{\widetilde{d}}_{2}^{2}$$

Similarly, as before we can demonstrate that:

45 $$\dot{\overline{V} }\le -{\overline{{\varvec{s}}} }^{T}{\varvec{K}}\overline{{\varvec{s}} }$$

**Fig. 3:**
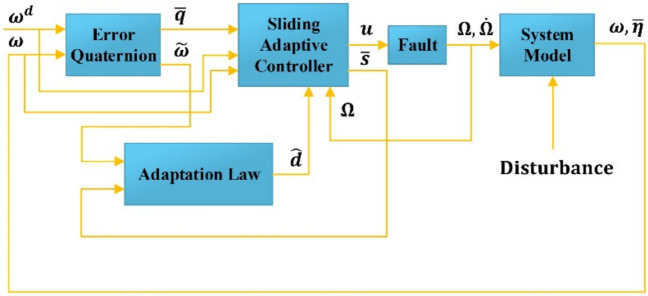
System schematic under proposed controller.

Thus, the system global boundedness inside the boundary layer is resulted^27^. The control system block diagram under the proposed control formula is depicted in Fig. 3. As is obvious after computing the control law, we have to transform it into the angular velocity and the angular acceleration of the reaction wheels. This is because the satellite’s inputs, according to $\left(1\right)$, are the reaction wheels angular momentum and their time derivatives.

## Simulation results

To evaluate the effectiveness and robustness of the devised controller, numerical simulation has been carried out. To approach reality, a satellite with the configuration as depicted in Fig. 1 is considered. The parameters of the satellite are considered to be as shown in Table 1. For ease of calculation, all products of inertia moments of the satellite are set as zero in the body frame. This implies that the body frame axes are oriented along the principal axes. In Table 1${I}_{xx}, {I}_{yy}, {I}_{zz}$ are the satellite’s principal moments of inertia, ${I}_{a}$ and ${I}_{t}$ are axial and transversal reaction wheels moments of inertial, respectively. $m$ is mass of each reaction wheel and $a$ is distance between reaction wheels center, and the center of body frame.

**Table 1:** Satellite parameters.

Parameter	Value (kgm^2^)
${I}_{xx}$	0.02
${I}_{yy}$	0.03
${I}_{zz}$	0.04
${I}_{a}$	$8\times {10}^{-4}$
${I}_{t}$	$4.33\times {10}^{-4}$
$m{a}^{2}$	$36\times {10}^{-4}$

For misalignment consideration in satellite reaction wheels, the misalignment angles are assumed to be as shown in Table 2. In this case, the satellite inertia matrix is derived as:

46 $${\varvec{J}}=\left[\begin{array}{ccc}{J}_{1}& {J}_{4}& {J}_{5}\\ {J}_{4}& {J}_{2}& {J}_{6}\\ {J}_{5}& {J}_{6}& {J}_{3}\end{array}\right] {\text{kgm}}^{2}$$

where ${J}_{1}$ to ${J}_{6}$ are as below:

**Table 2:** Satellite misalignment angles.

Angle	Value (rad)
${\alpha }_{1}$	$\pi /3$
${\alpha }_{2}$	$\pi /4$
${\alpha }_{3}$	$\pi /6$
${\beta }_{1}$	$\pi /4$
${\beta }_{2}$	$\pi /5$
${\beta }_{3}$	$\pi /7$

47 $$\begin{aligned}{J}_{1}&={I}_{xx}+{I}_{a}\left({{\cos}}^{2}\left({\beta }_{1}\right){{\cos}}^{2}\left({\alpha }_{1}\right)+{{\sin}}^{2}\left({\alpha }_{3}\right){{\cos}}^{2}\left({\beta }_{3}\right)+{{\sin}}^{2}\left({\beta }_{2}\right){{\cos}}^{2}\left({\alpha }_{2}\right)\right)\\ &\quad+{I}_{t}\left({{\sin}}^{2}\left({\beta }_{1}\right)+{{\cos}}^{2}\left({\beta }_{1}\right){{\sin}}^{2}\left({\alpha }_{1}\right)+{{\cos}}^{2}\left({\alpha }_{3}\right){{\cos}}^{2}\left({\beta }_{3}\right)+{{\sin}}^{2}\left({\beta }_{3}\right)+{{\cos}}^{2}\left({\beta }_{2}\right)+{{\sin}}^{2}\left({\beta }_{2}\right){{\sin}}^{2}\left({\alpha }_{2}\right)\right)+2m{a}^{2}\end{aligned}$$

48 $$\begin{aligned}{J}_{2}&={I}_{yy}+{I}_{a}\left({{\cos}}^{2}\left({\beta }_{2}\right){{\cos}}^{2}\left({\alpha }_{2}\right)+{{\cos}}^{2}\left({\alpha }_{1}\right){{\sin}}^{2}\left({\beta }_{1}\right)+{{\sin}}^{2}\left({\alpha }_{3}\right){{\sin}}^{2}\left({\beta }_{3}\right)\right)\\ &\quad+{I}_{t}\left({{\sin}}^{2}\left({\beta }_{2}\right)-{{\cos}}^{2}\left({\beta }_{2}\right){{\sin}}^{2}\left({\alpha }_{2}\right)+{{\cos}}^{2}\left({\beta }_{1}\right)+{{\sin}}^{2}\left({\beta }_{1}\right){{\sin}}^{2}\left({\alpha }_{1}\right)+{{\cos}}^{2}\left({\alpha }_{3}\right){{\sin}}^{2}\left({\beta }_{3}\right)+{{\cos}}^{2}\left({\beta }_{3}\right)\right)+2m{a}^{2}\end{aligned}$$

49 $$\begin{aligned}{J}_{3}={I}_{zz}+{I}_{a}\left({{\cos}}^{2}\left({\alpha }_{3}\right)+{{\sin}}^{2}\left({\alpha }_{1}\right)+{{\sin}}^{2}\left({\alpha }_{2}\right)\right)+{I}_{t}\left({{\sin}}^{2}\left({\alpha }_{3}\right)+{{\cos}}^{2}\left({\alpha }_{1}\right)+{{\cos}}^{2}\left({\alpha }_{2}\right)\right)+2m{a}^{2}\end{aligned}$$

50 $$\begin{aligned}{J}_{4}&={I}_{a}\left({{\cos}}^{2}\left({\alpha }_{1}\right){\sin}\left({\beta }_{1}\right){\cos}\left({\beta }_{1}\right)-{{\cos}}^{2}\left({\alpha }_{2}\right){\sin}\left({\beta }_{2}\right){\cos}\left({\beta }_{2}\right)+{{\sin}}^{2}\left({\alpha }_{3}\right){\sin}\left({\beta }_{3}\right){\cos}\left({\beta }_{3}\right)\right)\\ &\quad+{I}_{t}(-{\sin}\left({\beta }_{1}\right){\cos}\left({\beta }_{1}\right)+{{\sin}}^{2}\left({\alpha }_{1}\right){\sin}\left({\beta }_{1}\right){\cos}\left({\beta }_{1}\right)+{\sin}\left({\beta }_{2}\right){\cos}\left({\beta }_{2}\right)-{{\sin}}^{2}\left({\alpha }_{2}\right){\sin}\left({\beta }_{2}\right){\cos}\left({\beta }_{2}\right)\\ &\qquad+{{\cos}}^{2}\left({\alpha }_{3}\right){\cos}\left({\beta }_{3}\right){\sin}\left({\beta }_{3}\right)-{\sin}({\beta }_{3}){\cos}({\beta }_{3}))\end{aligned}$$

51 $$\begin{aligned}{J}_{5}&={I}_{a}\left(-{\sin}\left({\alpha }_{1}\right){\cos}\left({\beta }_{1}\right){\cos}\left({\alpha }_{1}\right)-{\sin}\left({\alpha }_{2}\right){\sin}\left({\beta }_{2}\right){\cos}\left({\alpha }_{2}\right)+{\sin}\left({\alpha }_{3}\right){\cos}\left({\alpha }_{3}\right){\cos}\left({\beta }_{3}\right)\right)\\ &\quad+{I}_{t}({\cos}\left({\alpha }_{1}\right){\cos}\left({\beta }_{1}\right){\sin}\left({\alpha }_{1}\right)+{\cos}\left({\alpha }_{2}\right){\sin}\left({\beta }_{2}\right){\sin}\left({\alpha }_{2}\right)-{\sin}({\alpha }_{3}){\cos}({\alpha }_{3}){\cos}({\beta }_{3}))\end{aligned}$$

52 $$\begin{aligned}{J}_{6}&={I}_{a}\left(-{\sin}\left({\alpha }_{1}\right){\sin}\left({\beta }_{1}\right){\cos}\left({\alpha }_{1}\right)+{\sin}\left({\alpha }_{2}\right){\cos}\left({\beta }_{2}\right){\cos}\left({\alpha }_{2}\right)+{\sin}\left({\alpha }_{3}\right){\sin}\left({\beta }_{3}\right){\cos}\left({\alpha }_{3}\right)\right)\\ &\quad+{I}_{t}({\cos}\left({\alpha }_{1}\right){\sin}\left({\beta }_{1}\right){\sin}\left({\alpha }_{1}\right)-{\cos}\left({\alpha }_{2}\right){\cos}\left({\beta }_{2}\right){\sin}\left({\alpha }_{2}\right)-{\sin}({\alpha }_{3}){\cos}({\alpha }_{3}){\sin}({\beta }_{3}))\end{aligned}$$

The effectiveness matrix and the additive bias term for the fault model is assumed as $${\varvec{E}}=\left[\begin{array}{ccc}0.85& 0& 0\\ 0& 0.9& 0\\ 0& 0& 0.8\end{array}\right]$$ and $$\Delta {\varvec{u}}=\left[\begin{array}{c}-0.01\\ -0.02\\ -0.05\end{array}\right] {\text{Nm}}$$, respectively. Moreover, the environmental disturbance is set to be $${\varvec{d}}={\left[\begin{array}{ccc}0.01{\sin}(0.4t)& 0.05{\cos}(0.5t)& 0.08{\cos}(0.7t)\end{array}\right]}^{T} {\text{Nm}}$$.

In order to calculate the control input, we assumed that there is no misalignment and fault in the actuators which means $\overline{{\varvec{D}} }$ and $\overline{{\varvec{E}} }$ are equal to identity matrix and $\overline{{\varvec{J}} }$ is calculated as below.

53 $$\overline{{\varvec{J}} }=\left[\begin{array}{ccc}{I}_{xx}+{I}_{a}+2{I}_{t}+2m{a}^{2}& 0& 0\\ 0& {I}_{yy}+{I}_{a}+2{I}_{t}+2m{a}^{2}& 0\\ 0& 0& {I}_{zz}+{I}_{a}+2{I}_{t}+2m{a}^{2}\end{array}\right] {\text{kgm}}^{2}$$

The controller gains are set as ${\varvec{C}}={{\varvec{I}}}_{3}$, ${\varvec{K}}={{\varvec{I}}}_{3}$, $b=2$, and the boundary layer thickness is considered as $\varphi =0.01$. The adaptation law gains are considered as ${k}_{0}=40$, ${k}_{1}=20$, ${k}_{2}=20$, and the initial values for ${\widehat{d}}_{0}, {\widehat{d}}_{1}, {\widehat{d}}_{2}$ are set as zero. The initial quaternion error for satellite is selected as $${\left[\begin{array}{cc}0.8986& 0.4 \end{array} \begin{array}{cc}-0.1& 0.15\end{array}\right]}^{T}$$, which the expected attitude is $$\left[\begin{array}{cc}1& 0\end{array} \begin{array}{cc}0& 0\end{array}\right]^{T}$$. In the following, the desirable angular velocity is $${{\varvec{\omega}}}_{{\varvec{d}}}={\left[\begin{array}{ccc}0.1{\cos}(t/40)& -0.1{\sin}(t/50)& -0.1{\cos}(t/60)\end{array}\right]}^{T} {\text{rad/s}}$$. In addition, the initial angular velocity of satellite and the reaction wheels are assumed to be zero.

### *Remark 3*

The saturation constraint of calculated control input is given as $Max\left(\left|{\varvec{u}}\right|\right)=0.12 {\text{Nm}}.$

**Fig. 4:**
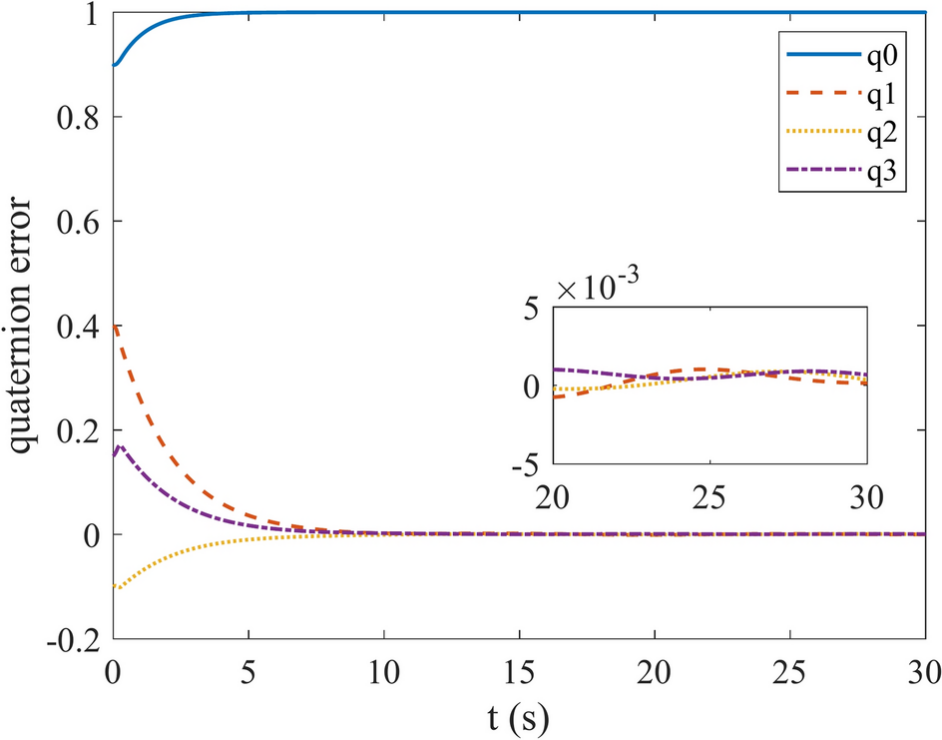
Quaternion error.

**Fig. 5:**
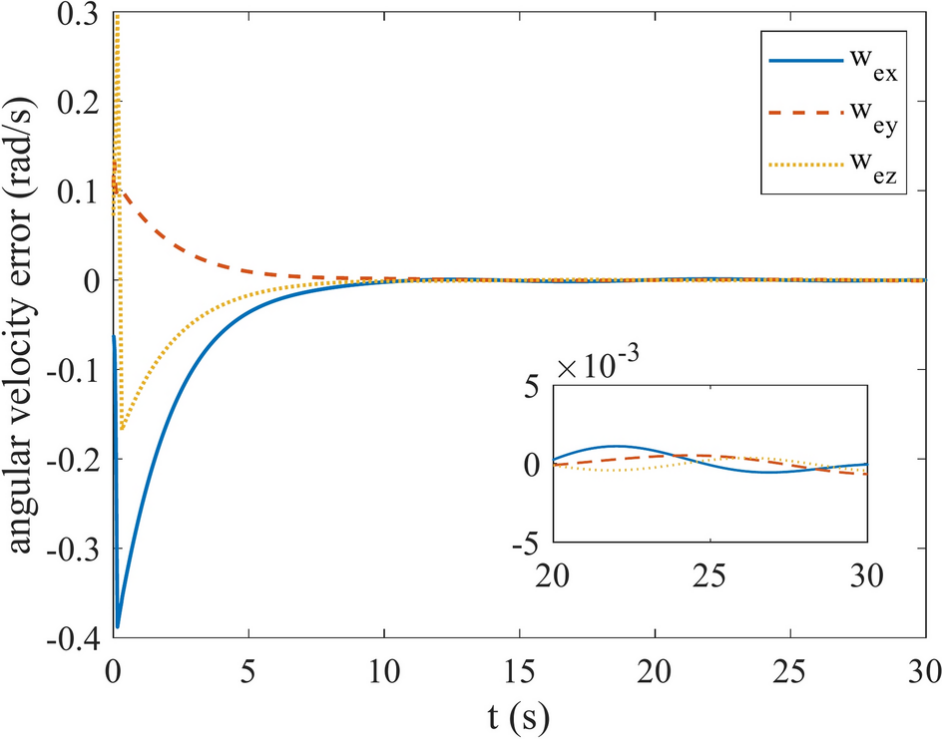
Angular velocity error.

**Fig. 6:**
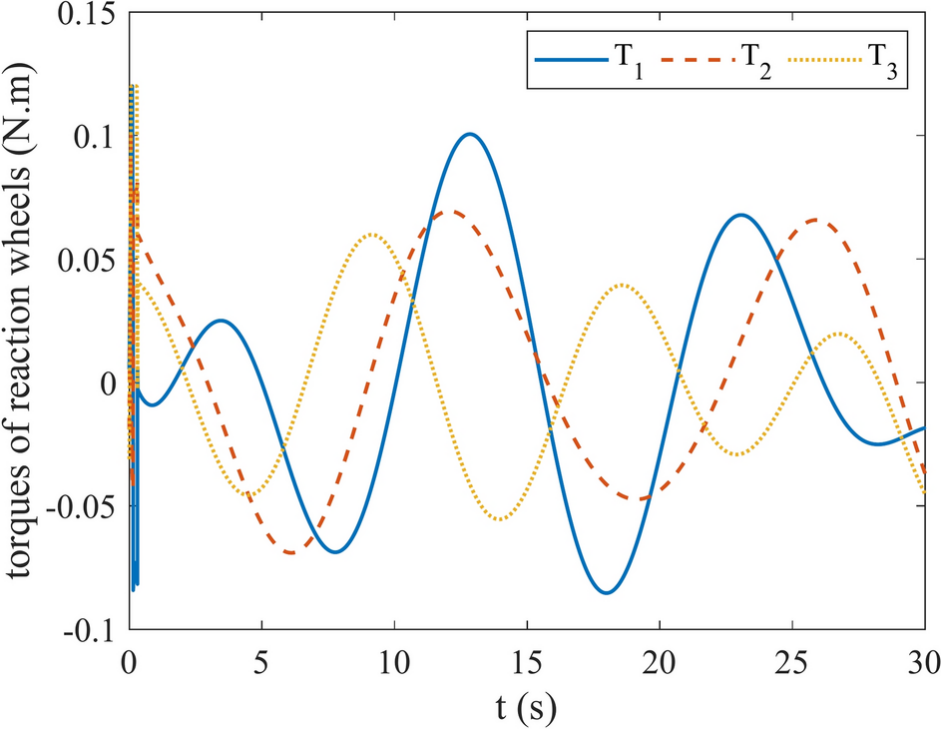
Torque of reaction wheels.

**Fig. 7:**
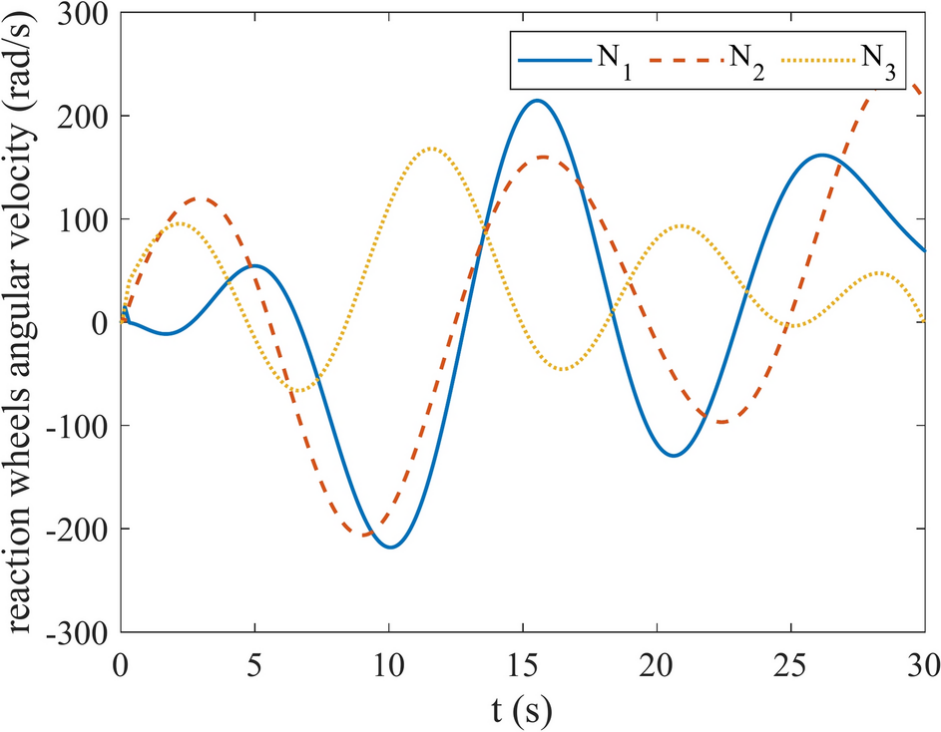
Angular velocity of reaction wheels.

Figures 4 and 5 show the quaternion error and angular velocity error, respectively. As is clear, both the quaternion error and angular velocity error converge to a small set containing the origin, which guarantees that the proposed controller has achieved desirable result. According to Fig. 4, it is concluded that satellite quaternion error converges to $${\left[\begin{array}{cc}1& 0\end{array} \begin{array}{cc}0& 0\end{array}\right]}^{T}$$ with 2% settling time of 7 s. Figures 6 and 7 depict the reaction wheels torque and angular velocity of the reaction wheels under the proposed controller. It can be seen that using a saturation function instead of a sign function is effective in reducing chattering. It is noticeable that the smaller the boundary layer value, the greater the chattering exists in the control input.

**Fig. 8:**
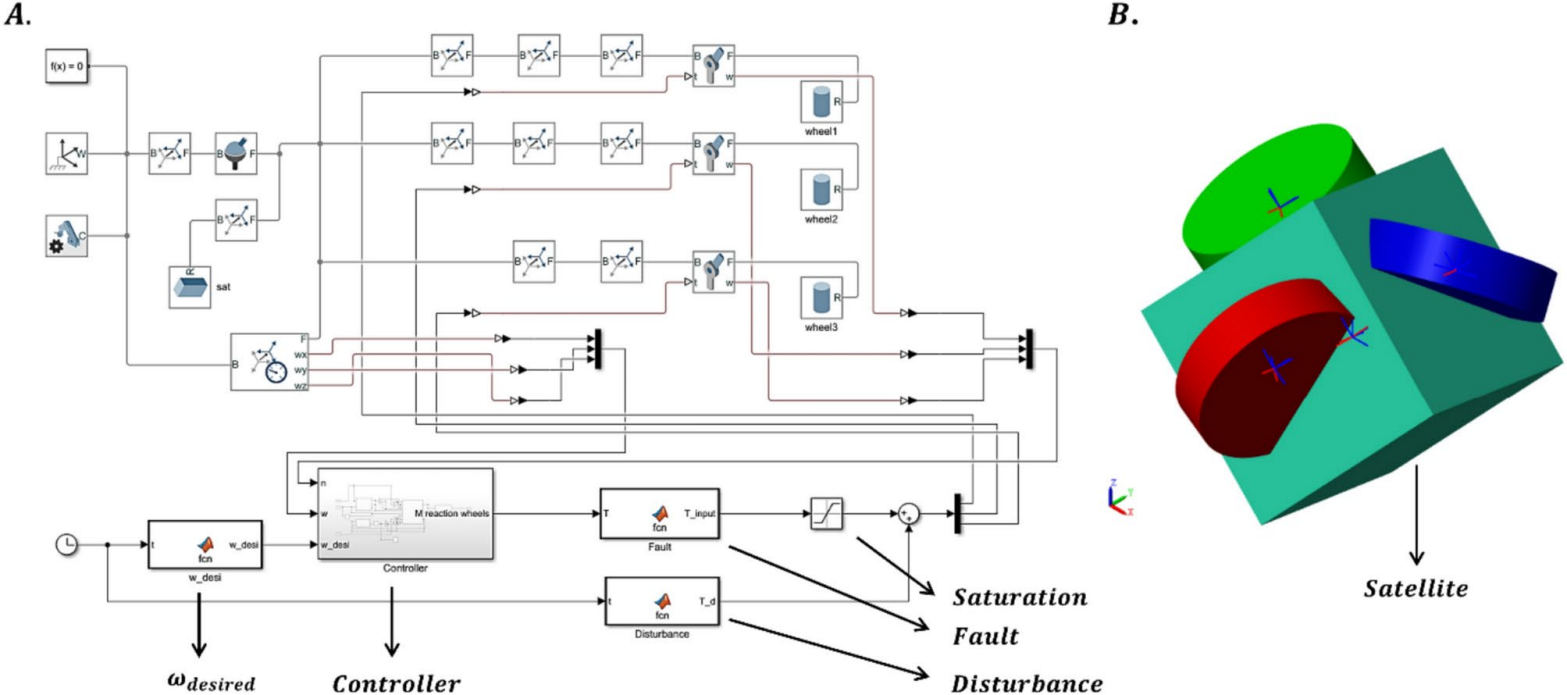
MATLAB Simscape Multibody environment. (**A**) Multibody block scheme. (**B**) Graphical representation of satellite in Mutlibody environment.

**Fig. 9:**
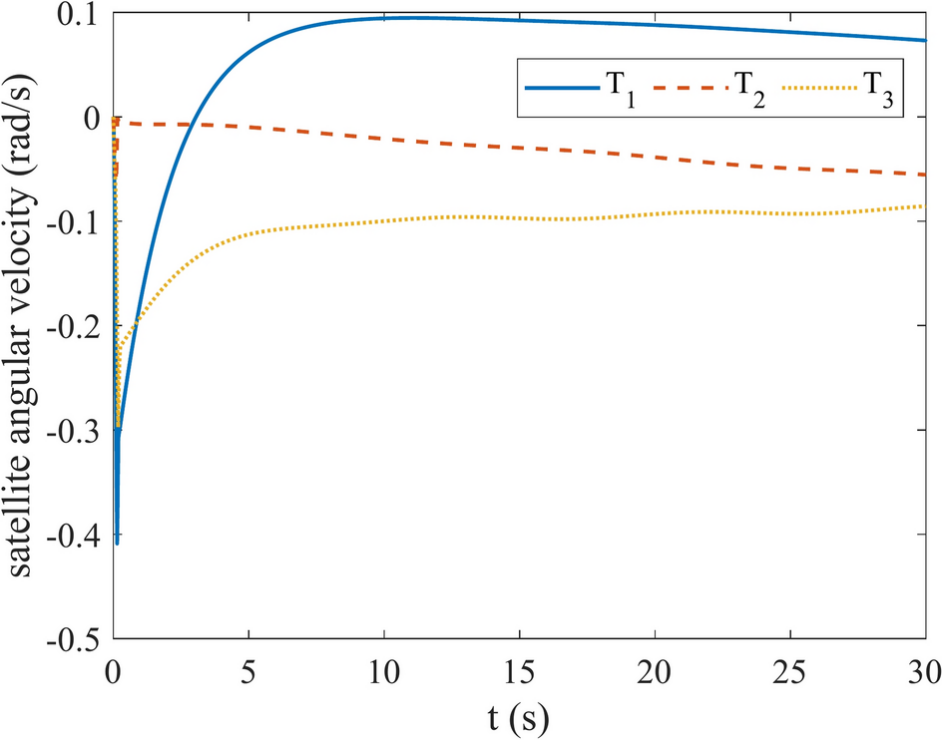
Satellite angular velocity in Multibody environment.

**Fig. 10:**
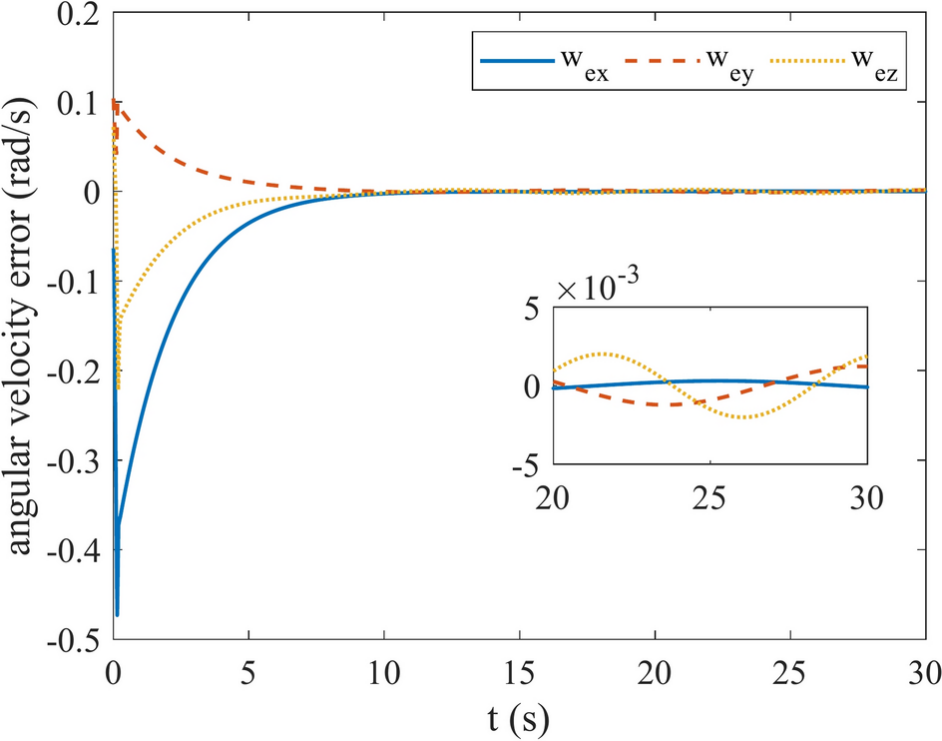
Angular velocity error in Multibody environment.

**Fig. 11:**
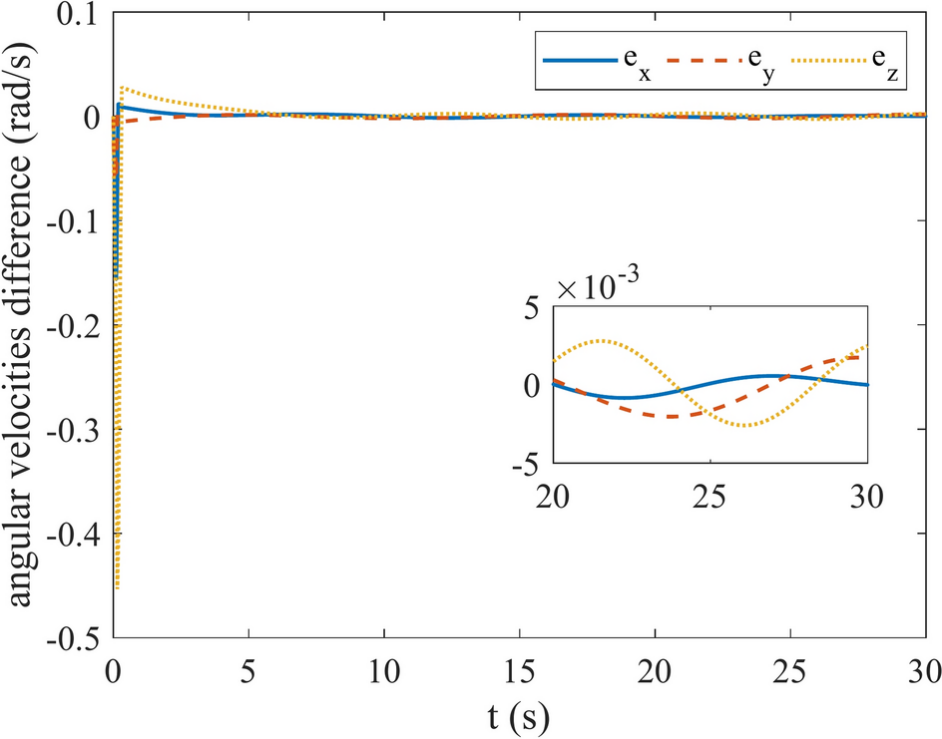
Verification error.

So as to verify the functionality of the propounded controller, the satellite is modeled in the MATLAB Multibody environment. Figure 8 shows details about modeled satellite in Simscape Multybody of MATLAB. In this environment we are able to model our satellite physically and then connect designed controller to that in order to evaluate our controller performance. In Fig. 8A the block diagram of a satellite with the controller is depicted. In Fig. 8B the graphical representation of satellite in multibody is showed. It can be seen that the satellite with three reaction wheels, which have misalignment, is modeled. At this case, the designed controller is connected to this model to control it ensuring that the satellite follows the desired angular velocity and attitude. Figures 9 and 10 show the angular velocity and error of angular velocity respectively. The difference between angular velocities of the model in the loop (MIL) system and the satellite modeled in Multibody environment is illustrated in Fig. 11. As is clear, the maximum verification error is about 0.004 rad/s. By noticing Figs. 9 and 11 maximum error in this analysis can be calculated which is less than 4%. Therefore, the proposed controller’ functionality is successfully verified and satisfies the predefined expectations.

**Fig. 12:**
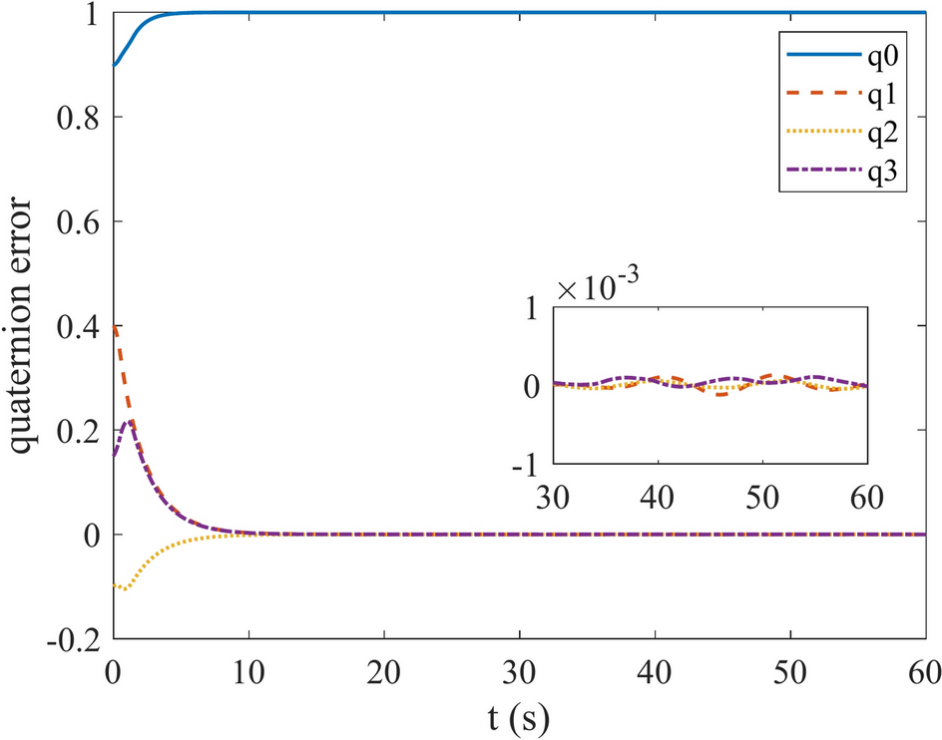
Quaternion error with 50% inertia uncertainty.

**Fig. 13:**
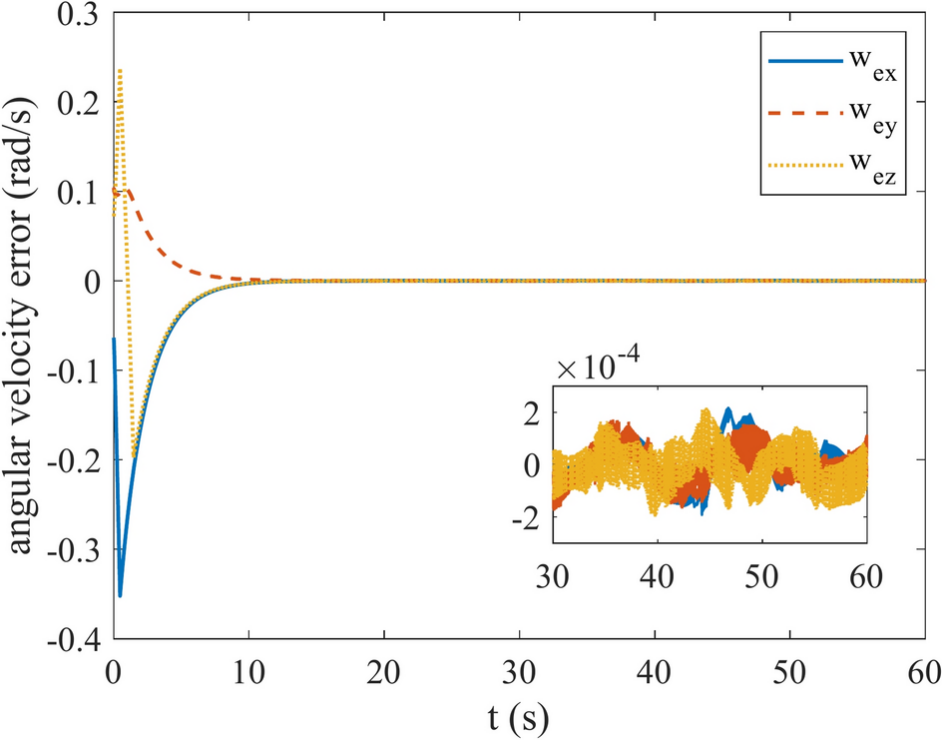
Angular velocity error under 50% inertia uncertainty.

**Fig. 14:**
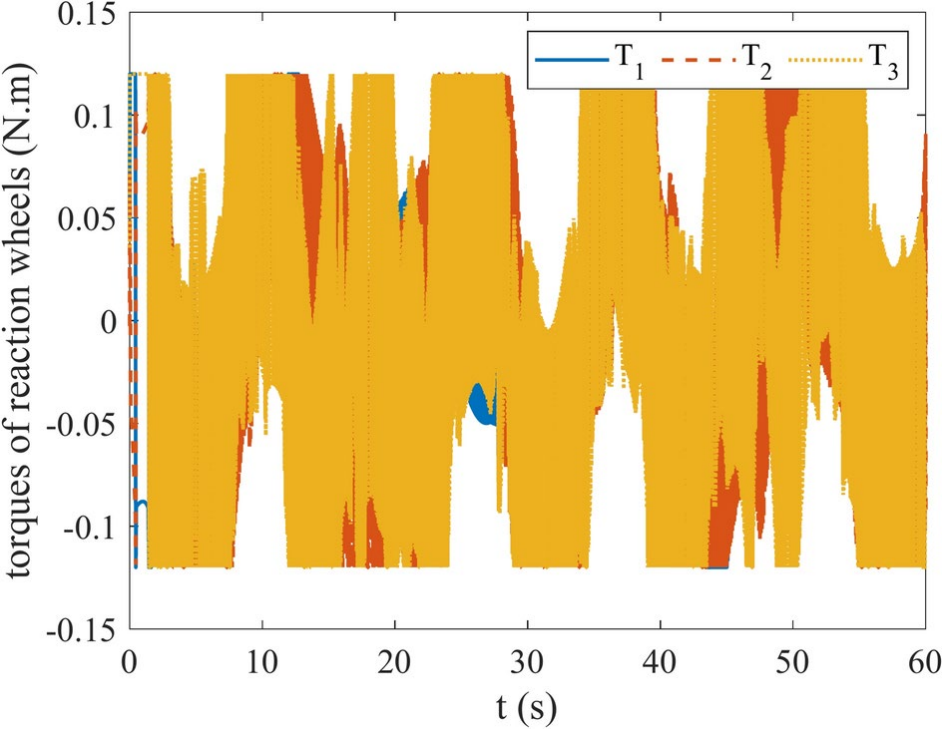
Reaction wheels torque generation under 50% inertia uncertainty.

For further examination of the satellite performance with proposed controller, at the first step, we simulate our model with more severe condition on inertia matrix uncertainty. Therefore, we consider satellite inertia matrix as (54) and introduce 50% uncertainty in satellite inertia. Other parameters and conditions remain as before. The purpose of simulating the model at this situation is to evaluate the controller’s capability in handling such a high inertia uncertainty. The simulated results are depicted in Figs. 12 and 13. As it is clear the quaternion error and angular velocity error of the satellite is in acceptable region but we can see high chattering in torques of reaction wheels. As shown in Fig. 14, saturation constraint in the torque of reaction wheels can be observed either.

54 $${\varvec{J}}=\left[\begin{array}{ccc}0.2& 0.01& 0.03\\ 0.01& 0.3& 0.05\\ 0.03& 0.05& 0.4\end{array}\right] {\text{kgm}}^{2}$$

**Fig. 15:**
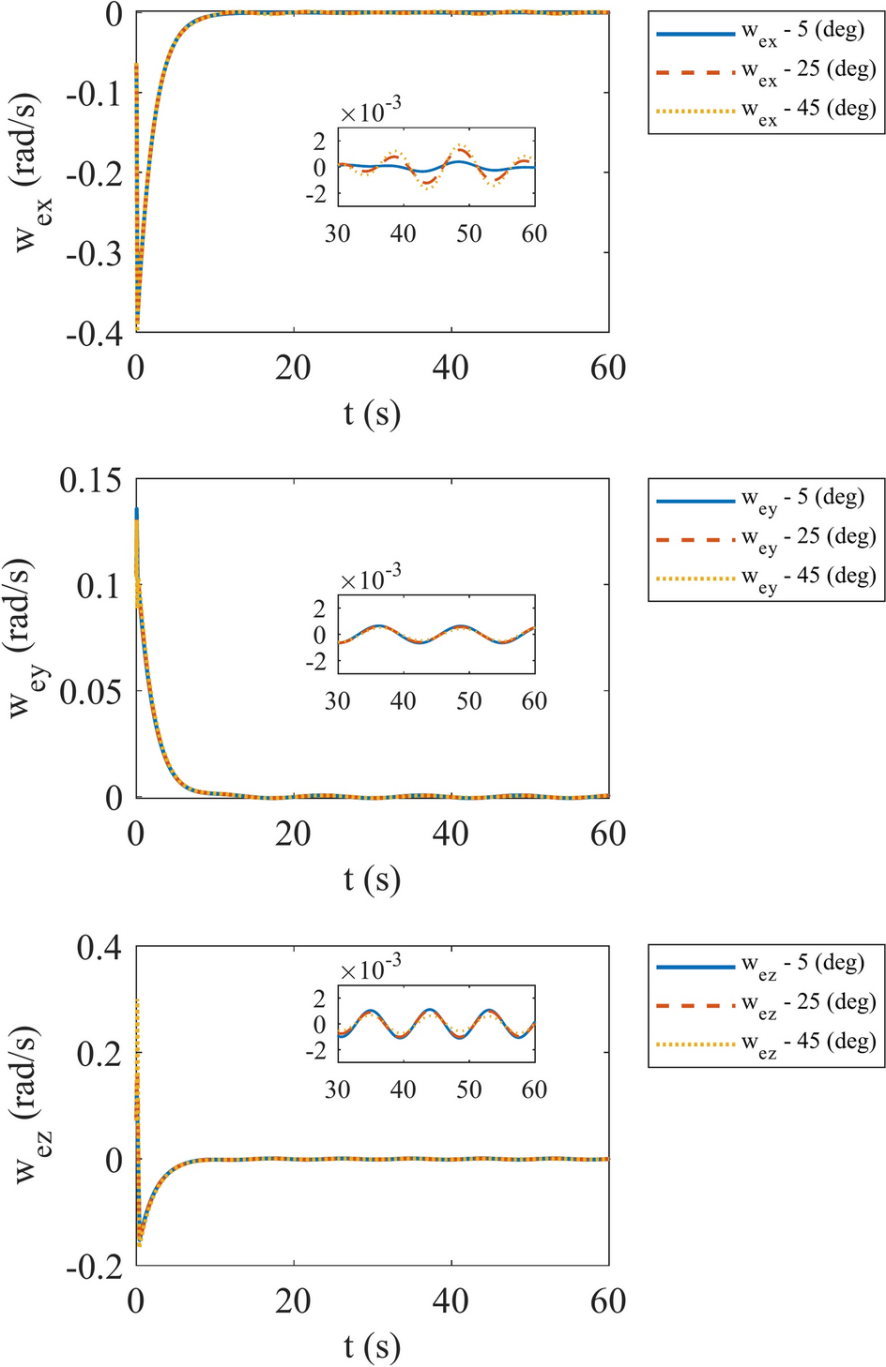
Angular velocity error with different misalignments.

**Fig. 16:**
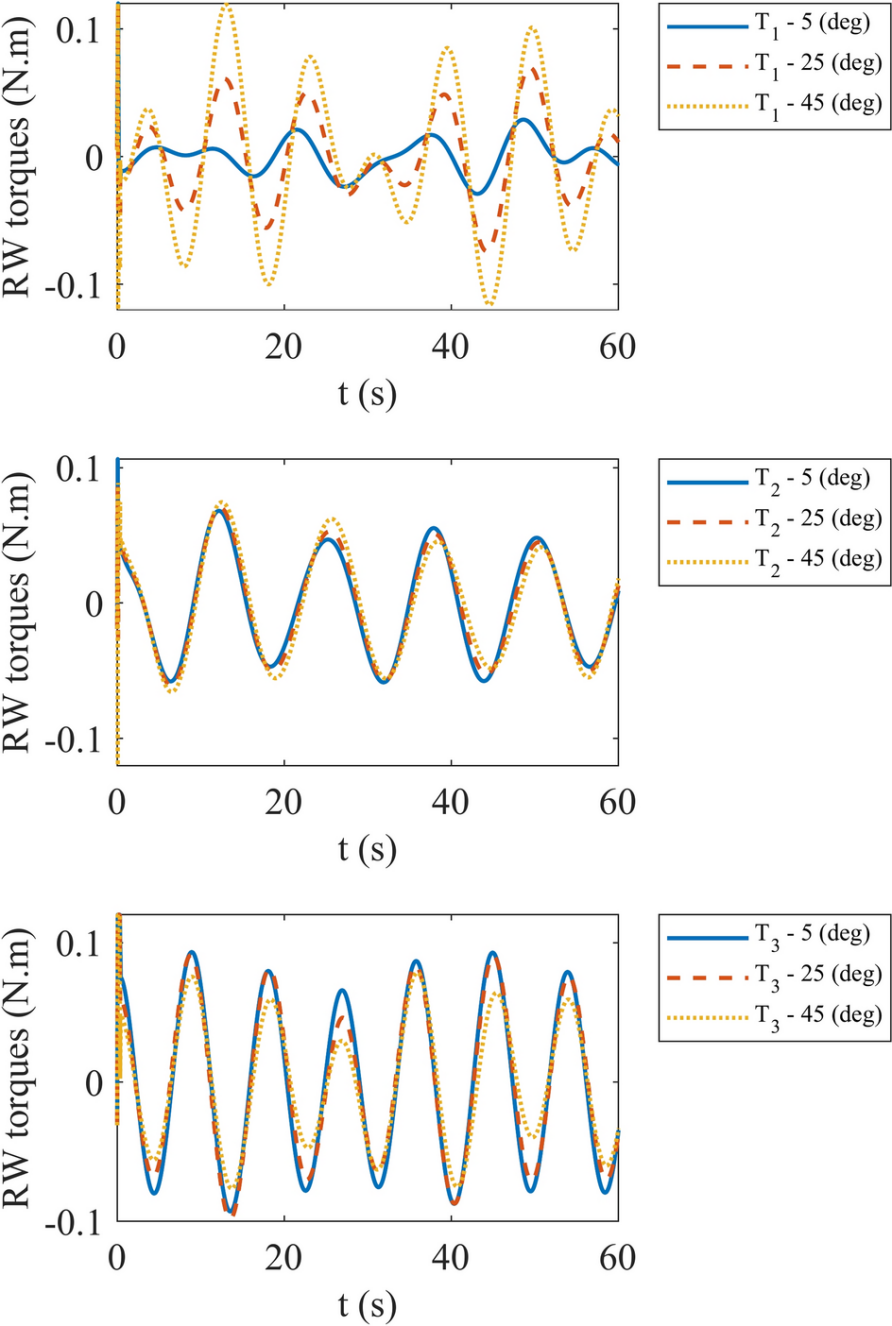
Reaction wheels torque with different misalignments.

In the second step we aim to evaluate the performance of the system with different misalignments. We assume that ${\alpha }_{i}, {\beta }_{i} (i={1,2},3)$ are same and simulate our satellite with misalignment values of 5°, 25° and 45° respectively. The angular velocity error at this case is depicted in Fig. 15. It can be mentioned that by increasing misalignment angle, X component of angular velocity error increases. The other components of angular velocity error are approximately near each other. By noticing the reaction wheels torque in Fig. 16, we can express that by increasing misalignment angle, torque of first reaction wheel increases while third reaction wheel torque decreases. Moreover, second reaction wheel torque exhibits variable behavior. It should be announced that by increasing misalignment angle over 45° reaction wheels saturate and controller can’t fulfil our expectations. The maximum torque of each reaction wheel at different misalignment angle is pointed out in Table 3.

**Table 3:** Maximum torque for each misalignment angle.

Misalignment (°)	Maximum torque (Nm)		
	*T*_1_	*T*_2_	*T*_3_
5	0.12	0.1063	0.12
25	0.12	0.0859	0.12
45	0.12	0.0887	0.12

**Fig. 17:**
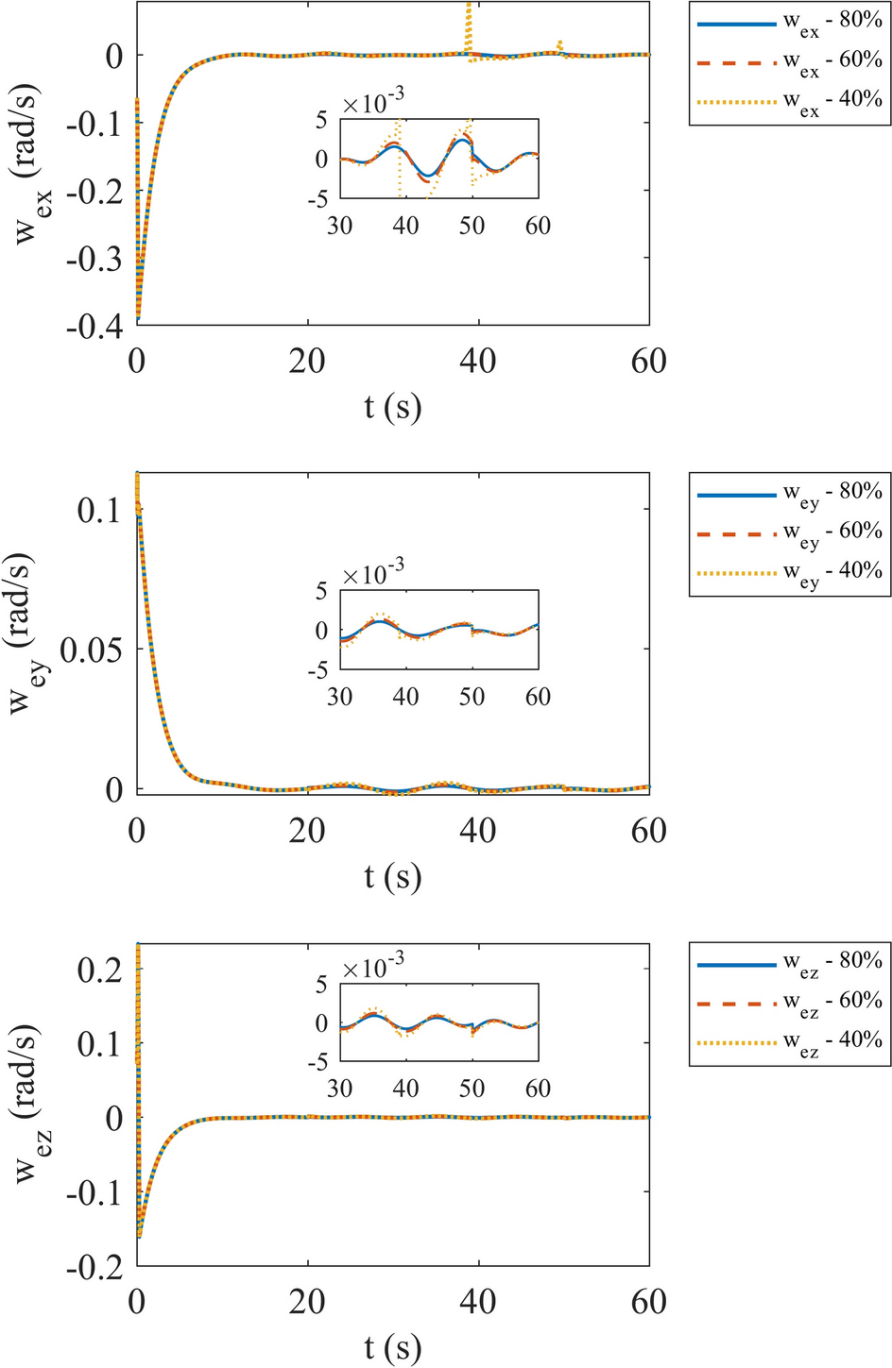
Angular velocity error with different faults.

**Fig. 18:**
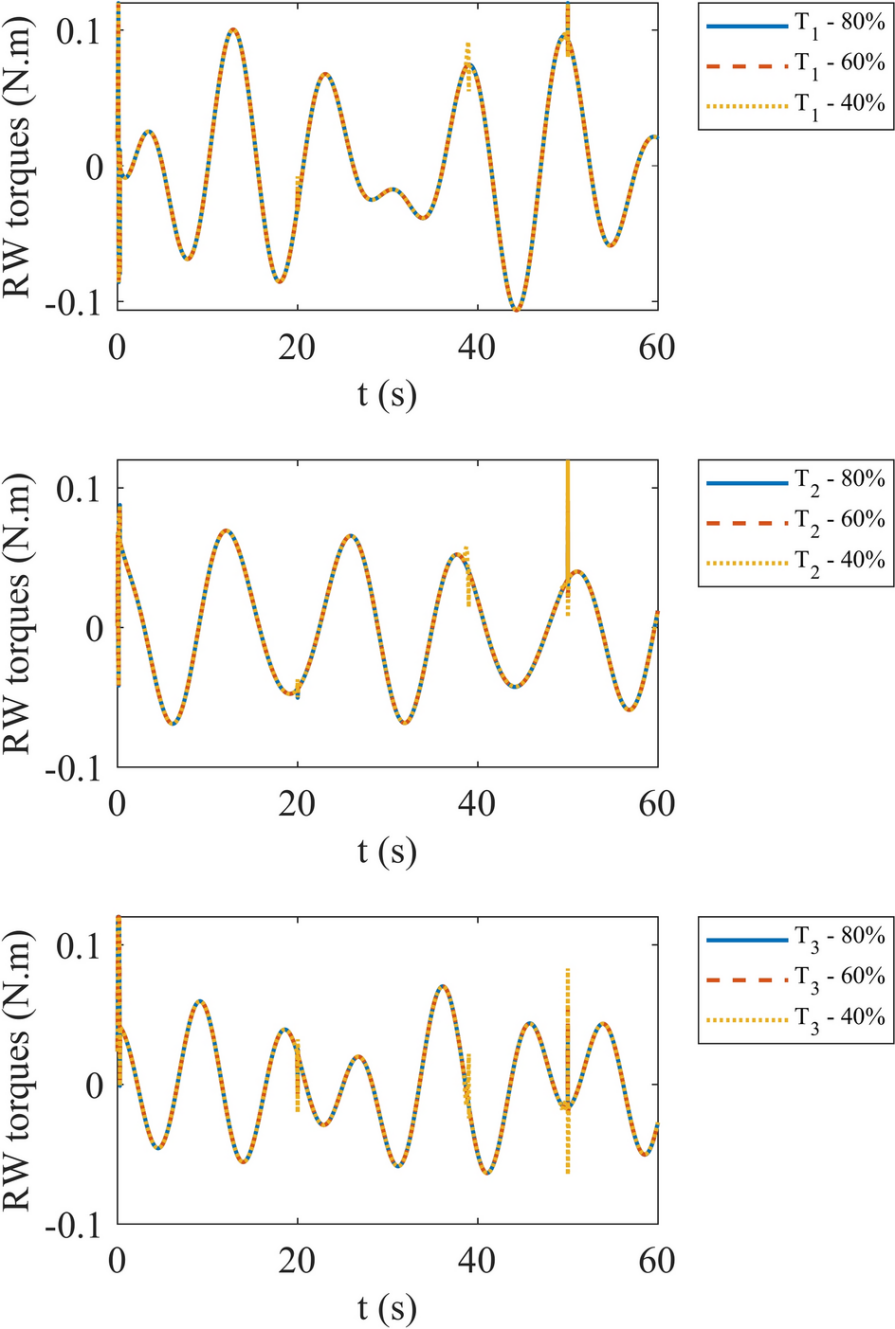
Reaction wheels torque with different faults.

In third step, evaluation of system with different faults is in consideration. We assume three different effectiveness matrices: $0.8{I}_{3}$, $0.6{I}_{3}$ and $0.4{I}_{3}$. By the way, we consider that fault occurs between 20 s and 50 s of simulation. The results of the system simulation in this case are demonstrated in Fig. 17. It is evident that by decreasing effectiveness matrix diagonal values, overall angular velocity error increases either. Figure 18 shows the reaction wheels torque for each effectiveness matrix value. According to Fig. 18 we can observe that decreasing values of effectiveness matrix cause higher peaks in start and end points of fault time happening. Moreover, the maximum torque of the reaction wheels in each situation is declared in Table 4.

**Table 4:** Maximum torque for each fault percentage.

Fault (%)	Maximum torque (Nm)		
	*T*_1_	*T*_2_	*T*_3_
80	0.12	0.0878	0.12
60	0.12	0.0906	0.12
40	0.12	0.12	0.12

## Conclusion

This paper proposes an adaptive sliding mode controller for the satellite attitude tracking with environmental disturbances. The designed controller can handle the satellite inertia matrix uncertainty, the actuator faults, and the reaction wheel misalignment. A key strength of the proposed controller is that it does not require knowledge of the upper bound values of uncertainties or environmental disturbances to generate the control input. For the defined problem, sliding surfaces were introduced, and the globally asymptotic stability of the entire system was demonstrated using the Lyapunov method. The controller successfully stabilized the tracking errors within a vicinity of the origin. The effectiveness and robustness of the proposed controller were demonstrated through simulation results, which highlighted the merits of the designed scheme. The simulation results showed that the quaternion error converges to $${\left[\begin{array}{cc}1& 0\end{array} \begin{array}{cc}0& 0\end{array}\right]}^{T}$$ with 2% settling time of 7 s and a saturation constraint of 0.12 Nm. Furthermore, to verify the controller’s performance, the system was modeled in MATLAB Multibody and integrated with the designed controller. The maximum verification error for the angular velocity errors was approximately 0.004 rad/s which was less than 4%.

## Data Availability

The datasets used during the current study are available from the corresponding author on reasonable request.
